# Identification and Characterization of Extrachromosomal Circular DNA in Human Placentas With Fetal Growth Restriction

**DOI:** 10.3389/fimmu.2021.780779

**Published:** 2021-12-21

**Authors:** Huan Yang, Jie He, Shuai Huang, Hongbing Yang, Qingjie Yi, Yuelan Tao, Miaomiao Chen, Xuemei Zhang, Hongbo Qi

**Affiliations:** ^1^ Department of Obstetrics, The First Affiliated Hospital of Chongqing Medical University, Chongqing, China; ^2^ Department of Obstetrics, Chongqing University Three Gorges Hospital, Chongqing, China; ^3^ Chongqing Key Laboratory of Maternal and Fetal Medicine, Chongqing Medical University, Chongqing, China; ^4^ China-Canada-New Zealand Joint International Research Laboratory of Reproduction and Development of Chinese Ministry of Education, Chongqing Medical University, Chongqing, China; ^5^ Department of Epidemiology and Health Statistics, School of Public Health and Management, Chongqing Medical University, Chongqing, China

**Keywords:** eccDNA, ecDNA, fetal growth restriction, placenta, Circle-seq, immunity

## Abstract

Many studies have confirmed that extrachromosomal circular DNAs (eccDNAs/ecDNAs) exist in tumor and normal cells independently of the chromosome and are essential for oncogene plasticity and drug resistance. Studies have confirmed that there are many eccDNAs/ecDNAs in maternal plasma derived from the fetus. Fetal growth restriction (FGR) is a pregnancy-related disease associated with high newborn morbidity and mortality. However, the characteristics and nature of eccDNAs/ecDNAs in FGR are poorly understood. This study aims to deconstruct the properties and potential functions of eccDNAs/ecDNAs in FGR. We performed circle-seq to identify the expression profile of eccDNAs/ecDNAs, analyzed by bioinformatics, and verified by real-time Polymerase Chain Reaction (PCR) combined with southern blot in FGR compared with the normal groups. A total of 45,131 eccDNAs/ecDNAs (including 2,118 unique ones) were identified, which had significantly higher abundance in FRG group than in normal group, and was bimodal in length, peaking at ~146bp and ~340bp, respectively. Gestational age may be one independent factor affecting the production of eccDNAs/ecDNAs, most of which come from genomic regions with high gene density, with a 4~12bp repeat around the junction, and their origin had a certain genetic preference. In addition, some of the host-genes overlapped with non-coding RNAs (ncRNAs) partially or even completely. Gene Ontology (GO) and Kyoto Encyclopedia of Genes and Genomes (KEGG) pathway enrichment analysis revealed that host-genes on the differentially expressed eccDNAs/ecDNAs (DEEECs/DEECs) were mainly enriched in immune-related functions and pathways. The presence of some ecDNAs were verified, and whose variability were consistent with the circle-seq results. We identified and characterized eccDNAs/ecDNAs in placentas with FGR, and elucidated the formation mechanisms and the networks with ncRNAs, which provide a new vision for the screening of new biomarkers and therapeutic targets for FGR.

## Introduction

Extrachromosomal circular DNAs(eccDNAs/ecDNAs) was first described by Alix Bassel more than 50 years ago (1), which widely exist in eukaryotic cells such as yeast, nematodes, ciliates, plants, and mammals (2–5), and has homology with chromosomal DNA. They have extensive heterogeneity in length, number, and origin due to different types and genetic backgrounds of cells and tissues (6). EccDNAs/ecDNAs are tissue-specific in mammals, which may lead to senescence and participate in gene compensation and intercellular communication. According to the size of the circular chromosome, we usually refer to the small circular DNA entities as eccDNAs (2, 7) and the large entity as ecDNAs (8–10). The latter typically contain oncogenes or drug resistance genes, which leads to oncogene amplification and is a powerful driving force for tumor heterogeneity (9, 11, 12). In addition, Dutta et al. found that eccDNAs can also produce small regulatory RNAs to regulate gene expression in a promoter-independent manner (13). Dennis Lo and his colleagues have identified and characterized fetal-derived eccDNAs in maternal plasma with a high abundance, indicating that it can be used as a potential serological marker for non-invasive prenatal diagnosis (14). However, the characteristics of eccDNAs/ecDNAs in the placenta are unknown, and the effect of cyclization on gene remodeling mediates pregnancy-related diseases is unclear.

Fetal growth restriction (FGR) refers to the failure of the fetus to reach its due growth potential in the maternal uterus due to various adverse reasons, which is a severe complication that endangers the safety of perinatal babies (15). The diagnostic criteria are that the fetal weight is lower than the 10th percentile for the same gestational age (16). Birth weight lower than the 3rd percentile is regarded as severe FGR, which is also directly related to severe adverse pregnancy outcomes of the fetus (17, 18). The pathogenesis of FGR is complex, with placental dysgenesis being an essential pathogenetic foundation (19). Therefore, we are intrigued by the nature and origin of eccDNAs/ecDNAs derived from the placenta with FGR.

In this study, we used Circle-Seq to extract, sequence, and locate extracellular cyclization elements in the placenta from the normal and severe FGR groups. To facilitate subsequent analysis, we divided the circular entities into eccDNAs and ecDNAs with the length of 100kb as the boundary. This study found that: (1) EccDNAs/ecDNAs is abundant and significant individual heterogeneity in the placenta, with higher abundance in the FGR group. (2) The formation of eccDNAs/ecDNAs is not a random incident and has some genetic preference, with microhomologous repeats as its main feature. (3) EccDNAs as a potential driver of FGR through immune signaling pathways and a complex network with non-coding RNAs (ncRNAs). We have validated by real-time Polymerase Chain Reaction (PCR) and Southern blot that the relevant ecDNAs are indeed present in the placenta. Our results provide an unprecedented view of these enigmatic molecules in the placenta during gestation, but one that remains naive.

## Materials and Methods

### Study Participants and Sample Collection

This study was approved by the Ethics Committee of the First Affiliated Hospital of Chongqing Medical University (No: 2019-136), Chongqing, China. Informed written consent was obtained from all patients. We recruited three pregnant women with Severe FGR and three healthy pregnant women in this study, whose detailed clinical characteristics of patients are shown in **Supplementary Table 1**. Severe FGR was defined as an estimated fetal weight below the 3rd percentile for gestational age and confirmed after birth (17, 18). All participants were singleton pregnancy and underwent cesarean section without any major pregnancy complications, such as premature labor or preeclampsia, gestational diabetes mellitus, renal disease, premature rupture of membranes, etc. Placental tissues were collected immediately after delivery, then snap-frozen in liquid nitrogen and stored at -80°C for preservation.

### Library Construction and Sequencing

#### 1) Sample DNA Extraction

Total genomic DNA was extracted from the placenta tissue samples according to the Cetyl trimethyl ammonium bromide (CATB), and its concentrations were measured using a NanoDrop Microvolume Spectrophotometer (Thermo Scientific). Qualified DNA samples (OD260/280 values between 1.8 and 2.0) were used for further experiments.

#### 2) Circular DNA Enrichment

For each sample, the remaining linear genomic DNA was removed by exonuclease V (New England Biolabs) following the manufacturer’s instructions to enrich circular DNA. After rolling circle amplification (RCA) of circular DNA as previously described (20), the resultant circular DNA was digested with MspI (New England Biolabs) according to the manufacturer’s instructions. Then the next step is to implement library sequencing.

#### 3) Illumina Sequencing

We used 1ug amplified Circular DNA for paired-end (PE) library construction using the NEBNext DNA Library Prep Master Mix set for Illumina (New England Biolabs). PE high-throughput sequencing (150 cycles) was performed according to the manufacturer’s protocol (Illumina), and 150bp (PE150) on both ends of the library was sequenced by Illumina Novaseq 6000(Genedenovo Bio, Guangzhou, China).

### Clean Reads Filtering

Reads obtained from the sequencing machines included raw reads containing adapters or low-quality bases, which would affect the following assembly and analysis. fastp software (21) was used to get high-quality clean reads according to four stringent filtering standards: (1) Removing reads containing adapters; (2) Removing reads containing more than 10% of unknown nucleotides; (3) Removing reads that are all A bases;(4) Removing low quality reads containing more than 50% of low quality (Q-value ≤ 20) bases.

### Cluster Identification and Analysis

The circular DNA analysis software Circle-map was used for eccDNAs/ecDNAs detection (20). The basic principle of Circle-map was used for discordant reads pair across the circular DNA interface to locate the position of the circular DNA interface. Then, the soft clipped read was used to determine the exact position of the circular DNA interface. However, both discordant reads pair and soft-clipped read can quantify the subsequent eccDNAs/ecDNAs. In addition, the software will also comprehensively consider the sequencing reads coverage of the eccDNAs/ecDNAs interval and the change of sequencing depth compared with the peripheral interval (theoretically, the sequencing depth of the eccDNAs/ecDNAs interval will increase) to judge the reliability of eccDNAs/ecDNAs. Circular DNA with a reserved length of < 100kb is defined as eccDNA, and the length of circular DNA between 100kb and 10M was identified as ecDNA for the subsequent analysis, respectively. The detected split reads in at least one sample are greater than or equal to one.

### Alignment With the Reference Genome

The software Burrows-Wheeler Aligner(BWA) (22) was used to align the clean reads to Ensembl release 98(Human genes, GRch38.p13). Picard Tools was used to sort the results and mark the repeated sequences, and BEDTools (23) was used to make genome coverage statistics.

### Analysis of Circular DNA Source Region and Related Coding Genes

To analyze eccDNAs/ecDNAs distribution in the whole-genome chromosome region, we split each chromosome into 50kb windows, counted the number of Circular DNA in each window, and showed it with a Manhattan chart. We mapped the sequenced base sequences with the whole-genome sequences and referred to the matching motifs as overlapping motifs. According to the source region of circular DNA, we annotated eccDNAs/ecDNAs. We divided the genome into 5 ‘ UTR, coding-exon, Exon plus, introns, 3’ UTR, Genic, Genic_ up/down 2000bp, CpG Island, CpG Island_ up/down 2000bp, transposons, Alu regions, and then annotated eccDNAs/ecDNAs according to which region it belongs. If eccDNAs/ecDNAs come from coding gene regions (including Exon, intron, gene_up2000 bp, and gene_down2000 bp), we referred to such coding genes as eccDNAs/ecDNAs-related genes. We annotated the coding genes related to eccDNAs/ecDNAs. The common and unique eccDNAs/ecDNAs-related genes was compared among samples, as long as eccDNAs/ecDNAs is detected in any sample in a certain treatment group in the genomic region where a certain gene is located, the gene will be judged as the eccDNAs/ecDNAs -related gene.

### GC Composition of eccDNAs/ecDNAs Analysis

BEDTools combined with an in-house algorithm were used to determine the median percent GC of each identified eccDNAs/ecDNAs and their up-stream and down-stream regions with the same length as themself.

### eccDNAs/ecDNAs Abundance Analysis

For all detected eccDNAs/ecDNAs, the sum of split reads and discordant reads pair of each eccDNAs/ecDNAs was regarded as the number of effective interface positions Tags supporting eccDNAs/ecDNAs, and based on which Tags per million (TPM) value of each eccDNAs/ecDNAs was calculated using the following formula: TPM= T*1000000/M (T: the number of influential interface positions Tags supporting an eccDNAs/ecDNAs; M: the total number of effective interface positions Tags of all eccDNAs/ecDNAs).

Based on the sequencing information, we used R to carry out Principal Component Analysis (PCA), using dimension reduction to study the distance relationship between samples. We also took the expression of each eccDNAs/ecDNAs in any two samples, calculated their Pearson correlation coefficient, and then visually displayed their correlation in the form of a heat map.

### Differential Analysis of Circular DNA Expression

The input data of eccDNAs/ecDNAs differential expression analysis were eccDNAs/ecDNAs tag count data, which was analyzed by edgeR software (24). The analysis was mainly divided into three parts: (1) normalized the tag count; (2) Calculated the probability of hypothesis testing (p-value) according to the model; (3) Multiple hypothesis testing and correction for obtaining FDR value (error detection rate). Based on the differential analysis results, we selected eccDNAs/ecDNAs with P-value<0.05 and |log2FC|>1 as the differentially expressed eccDNAs/ecDNAs (DEEECs/DEECs), for which Gene Ontology (GO) and Kyoto Encyclopedia of Genes and Genomes (KEGG) enrichment analysis of related host-genes were consequently carried out.

### Functional and Pathway Enrichment Analysis of Circular DNA Related Genes

We performed GO and KEGG pathway enrichment analysis by using the “Clusterprofiler” R package to investigate the biological characteristics of these DEEECs/DEECs related host-genes.

### Junctional Motifs of Circular DNA Analysis

To explore the motif patterns on both sides of the eccDNAs/ecDNAs junction, we located the base composition of the up-stream and down-stream 20bp at the start and end positions of each eccDNAs/ecDNAs. The 20bp up-stream and down-stream of these eccDNAs/ecDNAs genome coordinates were output to BED file, and then the function “BEDTools getFasta” was used to extract all relevant sequences (23), which were further analyzed by R (14, 25).

### Circular DNA Related TEs and ncRNAs Analysis

To investigate the relationship of circular DNA with transposon elements (TEs) and ncRNAs, we downloaded annotations for TEs, lncRNAs, miRNAs, snoRNAs from Ensembl release 98 (Human genes, GRCh38.p13), and circRNAs from circBase (26), respectively. Then, the coordinates of TEs and ncRNAs were compared with that of eccDNAs/ecDNAs using the R/Bioconductor GenomicRanges and the SplicingTypesAnno packages.

### Experimental Validation

Outward PCR oligonucleotides were designed on NCBI and Primer5 and the DEEECs/DEECs were targeted to design primers that can produce products across-junction (**Supplementary Table 2**). Total genomic DNA from 6 normal and 6 FGR placentas (screening criteria as above) was obtained by CTAB. Linear genomic DNA was digested with restriction exonuclease V. The remaining looped elements were PCR amplified using specific primers across the junction, and the product was loaded on a 2% agarose gel for Southern bolt electrophoresis.

### Statistical Analysis

SPSS 26.0 statistical software was used for statistical description and analysis with a=0.05 as the test level. Statistical descriptions were expressed as mean ± standard (*x̄* ± *s*) deviation. The statistically significant differences using the independent-sample t-test. P < 0.05 was considered statistically significant.

## Results

### The Landscape of Extrachromosomal Circular DNA

**Figure 1:**
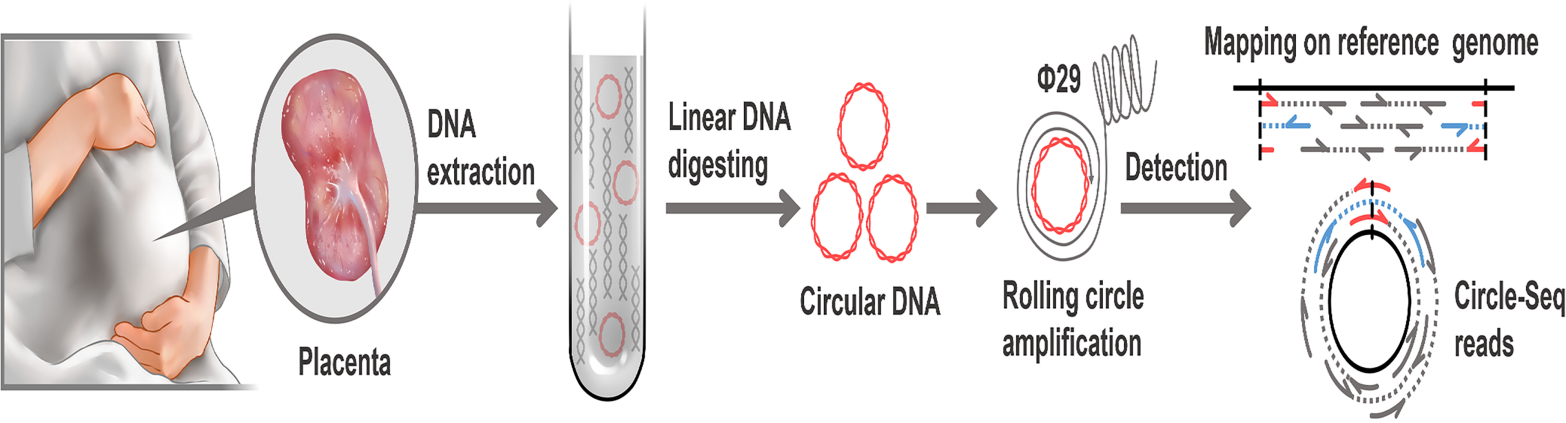
Workflow of eccDNAs/ecDNAs identification in placentas. Placentas were collected after elective deliveries. Total DNA from the placenta including linear and circular was extracted. linear DNA was digested off using exonuclease V. After the RCA of eccDNAs/ecDNAs. Then paired-end sequencing, localization and detection of eccDNAs/ecDNAs with paired-end reads (concordant reads, gray; discordant reads, blue. soft-clipped, split, red). Sequencing data were processed using bioinformatics for identification.

**Figure 2:**
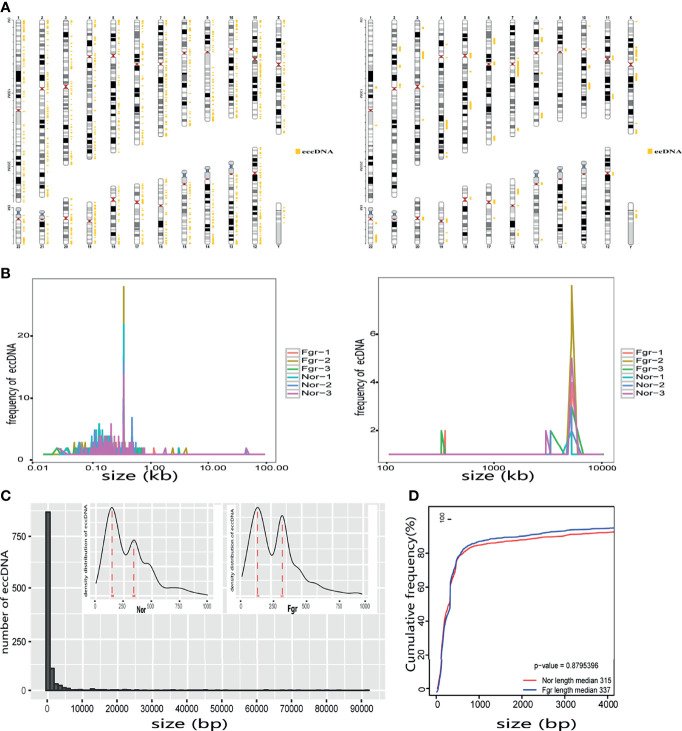
Expression profiles of eccDNAs/ecDNAs between FGR and normal groups. **(A)** Some eccDNAs/ecDNAs are shared between FGR and normal groups. **(B)** Hierarchical clustering of eccDNAs/ecDNAs profiles from six samples. **(C)** Heatmap showing the DEEECs/DEECs between FGR and normal groups.

**Figure 3:**
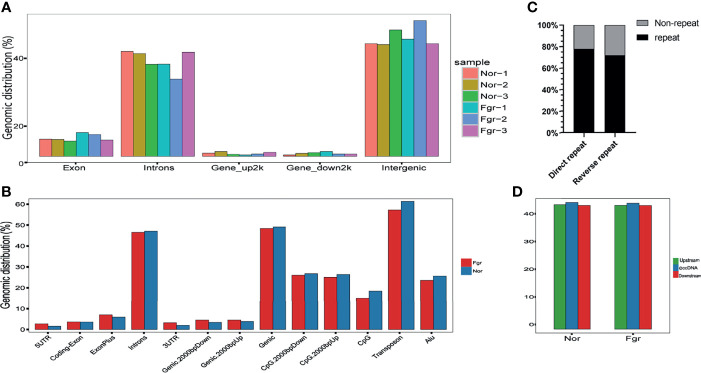
Size distribution of placental-derived eccDNAs/ecDNAs and their location on different chromosomes. **(A)** The distribution of eccDNAs/ecDNAs in different chromosomes. **(B)** Size profiling of total eccDNAs/ecDNAs in the placentas. **(C)** The length density distribution of eccDNAs of FGR and normal groups. There are three peaks at 146 bp, and 340 bp, respectively. **(D)** Cumulative frequency plot of eccDNAs in FGR and normal placentas. Sizes were capped at 4000bp for both groups of molecules.

Purification, enrichment, and detection of eccDNAs/ecDNAs from placental samples were performed in four steps **(** **Figure 1** **)**. A total of 41,247 eccDNAs and 3,884 ecDNAs were found in 6 samples, of which the number of unique eccDNAs and ecDNAs was 1,981 and 207, respectively (**Table 1**). We discovered that these circular elements were highly dynamic, and the result of Veen analysis in the normal and the FRG groups revealed only 188 eccDNAs and 52 ecDNAs overlaps (**Figures 2A, B** ***)***. We found that circular DNA can derive from any chromosome, and surprisingly chromosome 17 contains 193 unique eccDNAs and 67 unique ecDNAs, which was more than any other chromosome (**Figure 3A** and **Supplementary Table 3**). The result may be related to the fact that chromosome 17 carries more coding genes than other chromosomes, especially those associated with DNA damage repair (e.g., *TP53*) (11, 27), consistent with the results previously detected in normal human muscles and blood (20). We found that the length of eccDNAs in the placenta varies greatly but concentrates upon 100 ~ 400bp with two peaks at ~146bp and ~340bp, respectively (**Figures 3B, C** **)**. The length of eccDNAs around the mononucleosome or polynucleosome, suggested that DNA wrap around one or more nucleosomes may contribute to extrachromosomal circular DNA formation (7, 28), in line with previous reports on the characteristics of eccDNAs in maternal plasma derived from fetuses (14). In addition, we also found that many AA/AT/TT dinucleotide repeats were around the junction of cyclic elements (**Supplementary Data 7A, B**). CpG islands were periodically acid-interrupted by AA/AT/TT dinucleotides, which were characteristic of sequences that preferentially assemble into nucleosomes (29).

**Table 1:** Summary of eccDNAs and ecDNAs in placental tissues sequencing and mapping to the Human genome.

	Nor	FGR
**Paired End Reads**	898540217	1146221441
**Pairs Aligned**	878340041	1122056699
**Total Reads**	1797080434	2292442882
**Uniquely Aligned Reads**	1678336536	2140369014
**Multi-Mapped Reads**	106742704	137926095
**Total EccDNAs/EcDNAs**	17525/1453	23722/2431
**Unique EccDNAs/EcDNAs**	1239/120	926/139

### EccDNAs/ecDNAs Derived From Genic and Intergenic Regions

**Figure 4:**
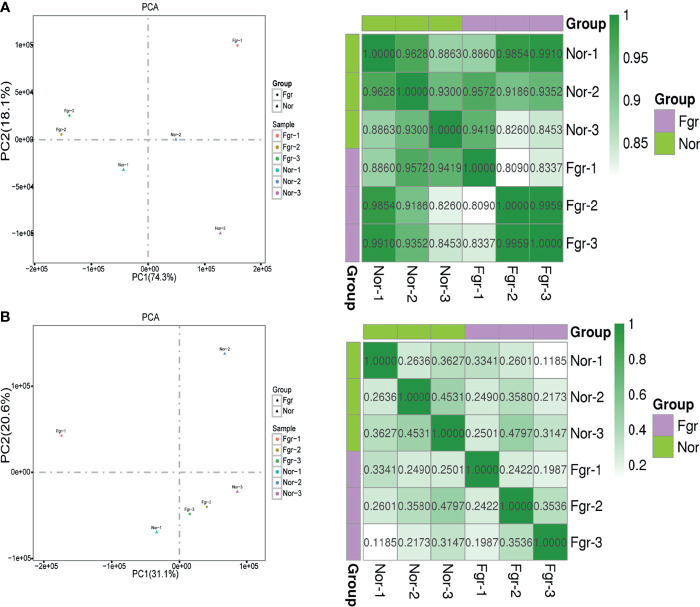
Properties of the loci that release eccDNAs. **(A)** The distribution of eccDNAs of each sample on whole-genome chromosome region. **(B)** Distribution of eccDNAs of FGR and normal groups in the indicated genomic region. **(C)** Percentage of eccDNAs with (black) or without (gray) 4~12bp direct or reverse repeats flanking junction locus at the genomic source. **(D)** Median percent GC content of eccDNAs and the genomic sequences of equal length upstream or downstream of the eccDNAs source loci.

To understand the origin of circular DNA in the genome and whether its characteristics affect its formation, we mapped the total population of eccDNAs/ecDNAs detected onto the whole-genome chromosome region, showing that 50.5% of eccDNAs and 50.8% of ecDNAs overlapped with gene regions (**Figure 4A** and **Supplementary Figure 1A**). Within the overlapping genomic motifs, they were concentrated in gene regions, CpG islands, introns, and TEs, respectively (**Figure 4B** and **Supplementary Figure 1B**), in a pattern similar to the observation in mice and non-pregnant human muscle (7, 30). The genes overlapped on those circular elements were highly dynamic, of which 52% (1030/1981) of eccDNAs covered partial gene fragments and 42% (87/207) of ecDNAs covered more than one complete gene (**Supplementary Data 1A, B**). Among these, 1030 unique eccDNAs partially overlapped with 939 genes, while the 87 unique ecDNAs overlapped with 3316 genes (**Supplementary Data 9A, B**). What is exciting is the large number of ecDNAs carried multiple genomes, forming super ecDNA [e.g., ecDNA(*chr10:62267596-65472354*) carrying 121 genes]. In addition, we found a preference for some genes being circular, with 5.9% (55/939) of host-genes derived from eccDNAs localized to more than three eccDNAs, while 0.81% (27/3316) of host-genes derived from ecDNAs had a similar profile. There were even many super genes positioned on circular DNAs, e.g., *PTPRN2*, which formed 11 different eccDNAs and was found in all 6 samples (**Supplementary Data 9A, B**). Notably, those circular elements from the X chromosome (41/92) tend to come from the gene region rather than the intergenic region compared with the Y chromosome (2/44) (**Supplementary Data 1A, B**). Therefore, eccDNAs/ecDNAs from the placenta is not completely random and shows a certain bias. Regrettably, we did not find a statistically significant difference in the genomic distribution of eccDNAs/ecDNAs derived between the normal and FGR groups (**Figure 3**).

### Motif Characteristic Around the eccDNAs/ecDNAs Junctions

We performed a BLAST analysis of 20bp upstream and downstream of the eccDNAs/ecDNAs junctions locus. Interestingly, we found that 77.7% of the eccDNAs had a direct repeat motif of 4~12bp upstream and downstream of the junction, and 71.5% of eccDNAs junction had a reverse repeating motif of 4~12bp (**Figure 4C**). Similar results were obtained for ecDNAs (**Supplementary Figure 1C**). The presence of “double-repeated trinucleotide sequences” on both sides of the junction was also found by using R (**Supplementary Data 7A, B**). These results suggest that microhomologous recombination may be an essential potential trigger for the promotion of double-stranded DNA cyclization.

### Effects of Pathological Factors and Gestational Age

**Figure 5:**
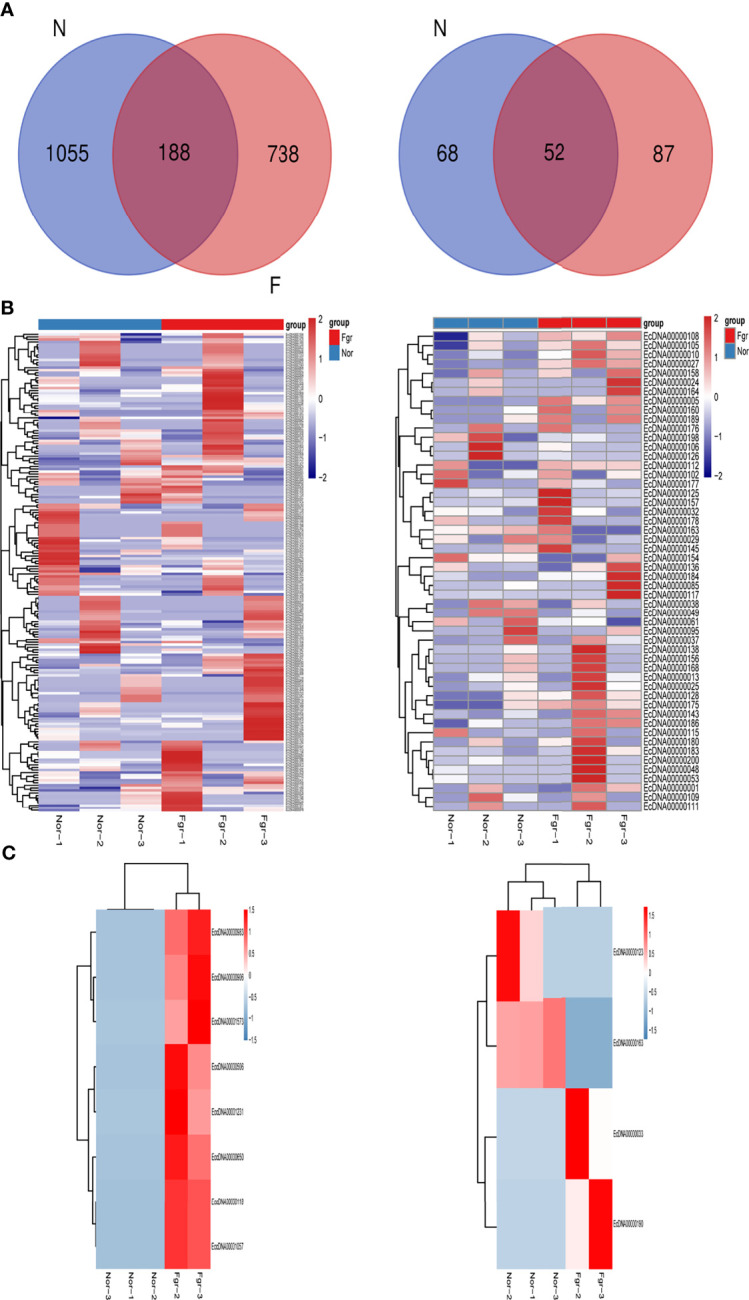
Correlation analysis between samples. **(A)** Principal component analysis and pearson correlation coefficient base on profile of eccDNAs of six samples. **(B)** Principal component analysis and pearson correlation coefficient base on profile of ecDNAs of six samples.

Next, we evaluated whether pathological factors affect circular DNA in placental tissue during pregnancy. The results showed that the total number of eccDNAs of FGR group (7907 ± 1514; n=3) was slightly higher than those in the normal group (5842 ± 479.4; n=3) (P=0.087) (**Supplementary Data 2A**). The content of total ecDNAs also increased in the FGR group (810.3 ± 187.9; n=3) compared with the normal group (484.3 ± 56.59; n=3) (P=0.045) (**Supplementary Data 2B**). No significant difference in the number of unique eccDNAs and ecDNAs were found between the two groups (unique eccDNAs: FGR group (353.7 ± 53.80; n=3) vs normal group (459.3 ± 67.16; n=3) (P=0.100) **(** **Supplementary Data 3A** **)**; unique ecDNAs: FGR group [64.33 ± 9.074; n=3) vs normal group (54.3 ± 4.619; n= 3) (P= 0.164) **(** **Supplementary Data 3B** **)]**. To clarify whether pathological factors affect the length of circular elements, we compared the length distribution of eccDNAs between the two groups, and there was no significant difference between the two groups (P=0.879) (**Figure 3D**). PCA and Pearson analysis was carried out between groups using R to investigate whether other relevant factors influenced eccDNAs/ecDNAs, and results showed that FGR1 was significantly away from the shared features within the group (**Figures 5A, B**).

### Biological Function of eccDNAs/ecDNAs

**Figure 6:**
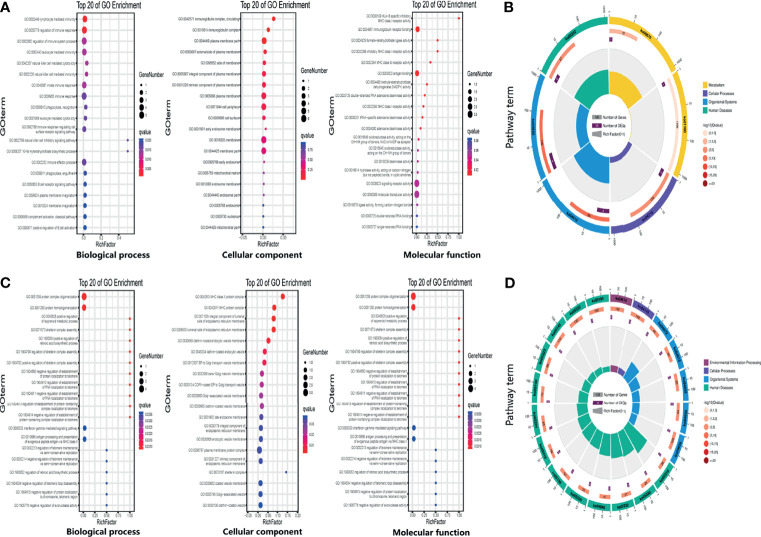
The enrichment map for differentially expressed host-genes on DEEECs/DEECs generated using GO and KEGG pathway analyses. **(A)** GO enrichment analyses of host-genes on DEEECs. **(B)** KEGG enrichment analyses of host-genes on DEEECs. **(C)** GO enrichment analyses of host-genes on DEECs. **(D)** KEGG enrichment analyses of host-genes on DEECs.

To investigate the potential biological functions of circular DNA in the placenta for disease pathogenesis, we performed a differential analysis of eccDNAs/ecDNAs between groups. The “ edgeR” package was used with the cut-off criteria of adjusted P-value<0.05 and |log2 FC|≥1. Results found that only one differential eccDNA (*Chr2: 90394483-90399164*) and one differential ecDNA (*Chr17: 23204862-26614801*), and their host-gene all annotated at the intergenic region, urging us to further re-analyze the sample information. Combined with the results of PCA, we excluded FGR1 and re-analyzed, which generated 8 DEEECs and 4 DEECs (**Figure 2C**). The results were compared with the reference genome using BWA software (22). We performed GO and KEGG pathway enrichment analysis by using the “Clusterprofiler” R package. The most enriched GO terms of host-genes on DEEECs were showed (**Figure 6A** and **Table 2**). The top 3 enriched terms of biological process were “lymphocyte-mediated immunity (GO:0002449, adjusted P = 0.001), regulation of immune response (GO:0050776, adjusted P = 0.002), and regulation of immune system process (GO:0002682, adjusted P = 0.012). The top 3 enriched terms of cellular component were “immunoglobulin complex, circulating (GO:0042571, adjusted P = 0.019), immunoglobulin complex (GO: GO:0019814, adjusted P = 0.029), and plasma membrane part (GO: GO:0044459, adjusted P = 0.029)”. The top 3 enriched terms of molecular function were “HLA-B specific inhibitory MHC class I receptor activity (GO: GO:0030109, adjusted P = 0.01), immunoglobulin receptor binding (GO: GO:0034987, adjusted P = 0.01), and formate-tetrahydrofolate ligase activity (GO:0004329, adjusted P =0.01)”. This host-gene significantly enriched GO terms on eccDNAs, which helps us further understand their role in FGR. Meanwhile, KEGG pathway analysis was further performed (**Figure 6B** and **Table 3**), in which host-genes were significantly enriched in “Antigen processing and presentation (ko04612, P = 3.85E-08), Natural killer cell-mediated cytotoxicity (ko04650, P = 4.43E-07), and Graft-versus-host disease (ko05332, adjusted P = 0.00025)”. We also analyzed the biological function of DEECs in the same way (**Figures 6C, D**). Interestingly, most of these different circular elements were in the FGR group.

**Table 2:** Top 5 enriched GO associated with host-genes on DEEECs.

GO		
	Go terms	Genes
**Cellular component**	Immunoglobulin complex, circulating	IGHV4-61; IGHV4-59
	Immunoglobulin complex	IGHV4-61; IGHV4-59
	Plasma membrane part	KIR2DL1; KIR3DL1; KIR2DL4; IGHV4-61; IGHV4-59
	External side of plasma membrane	IGHV4-61; IGHV4-59
	Side of membrane	IGHV4-61; IGHV4-59
**Molecular function**	HLA-B specific inhibitory MHC class I receptor activity	KIR3DL1
	Immunoglobulin receptor binding	IGHV4-61; IGHV4-59
	Formate-tetrahydrofolate ligase activity	MTHFD1L
	Inhibitory MHC class I receptor activity	KIR3DL1
	MHC class Ib receptor activity	KIR2DL4
**Biological process**	Lymphocyte mediated immunity	KIR3DL1; KIR2DL4; IGHV4-61; IGHV4-59
	Regulation of immune response	KIR2DL1; KIR3DL1; KIR2DL4; GHV4-61; IGHV4-59
	Regulation of immune system process	KIR2DL1; IR3DL1; KIR2DL4; IGHV4-61; IGHV4-59
	Leukocyte mediated immunity	KIR3DL1; KIR2DL4; IGHV4-61; IGHV4-59
	Natural killer cell mediated cytotoxicity	KIR3DL1; KIR2DL4

**Table 3:** Top 5 enriched KEGG pathways associated with host-genes on DEEECs.

KEGG		
Kegg_class	Pathway	Genes
**Immune system**	Antigen processing and presentation	KIR2DL1; KIR3DL1; KIR2DS4; KIR2DL4
**Immune system**	Natural killer cell mediated cytotoxicity	KIR2DL1; KIR3DL1; KIR2DS4; KIR2DL4
**Immune diseases**	Graft-versus-host disease	KIR2DL1; KIR3DL1
**Cell growth and death**	Cellular senescence	KIR2DL1; KIR2DL4
**Metabolism of cofactors and vitamins**	One carbon pool by folate	MTHFD1L

Studies have shown that ncRNAs play a significant role in FGR (31). We compared the genomic coordinates of eccDNAs/ecDNAs with ncRNAs using R/Bioconductor GenomicRanges and SplicingTypesAnno software package. Results revealed that 12.6% (250/1981) of the eccDNAs overlapped with the coordinates of 199 unique lncRNAs, and 0.05% (1/1981) of the eccDNAs overlapped with the coordinates of 22 unique tRNAs (**Supplementary Data 11A**). The same analysis uncovered that 40.6% (84/207) of ecDNAs overlapped with the coordinates of 1288 unique lncRNAs, 20.8% (43/207) of ecDNAs overlapped coordinates of 184 unique miRNAs, and 14.9% (31/207) of ecDNAs overlapped coordinates of 57 unique snoRNAs (**Supplementary Data 11B**). The results indicate that the function of these cyclic elements may be associated with lncRNA, tRNA, miRNA, snoRNA, etc., and some studies have demonstrated that under the conditions of stress, the expression of ncRNAs may change and participate in the progression of FGR (32, 33).

Lanciano et al. reported that eccDNAs were related to TEs activity (25, 34). In addition, it was recently found that the Ty1 subfamily of long terminal repeat sequence (LTR) retrotransposons was overexpressed in yeast eccDNAs (35). We further analyzed the correlation of TEs with eccDNAs/ecDNAs and found a high overlapping of 61.38% (**Figure 3B** and **Supplementary Table 4**). TEs is a movable DNA sequence (36) and contains a large number of repeating elements, which may contribute to the generation of eccDNAs/ecDNAs and drive tumor evolution and genetic heterogeneity (8). The high overlapping of ecDNAs/ecDNAs in the placenta annotated with the TEs is worthy to investigate.

Iparraguirre et al. proposed that circular DNA potentially forms transcripts analogous to circRNAs (37). We mapped all sequences of eccDNAs/ecDNAs to the database downloaded from circBase (26). 11,148 circRNAs were found to partially even all overlap with the circular DNA, among which the coordinates of ecDNA (*Chr 6:57345464-60256901*) could highly overlap with multiple circRNAs [e.g., *hsa_circ_0076892*: *Chr6:57317623-60430572* (93.5%) and *hsa_circ_0076899*: *Chr6: 57324200-60425439* (93.9%)] (**Supplementary Table 5**). Moreover, the vast majority of ecDNAs coordinates were are completely overlapped with their corresponding circRNAs. In light of this, we also attach a high priority to the possibility that eccDNAs/ecDNAs influences disease pathogenesis through the direct transcript form of circRNAs.

### Experimental Validation

**Figure 7:**
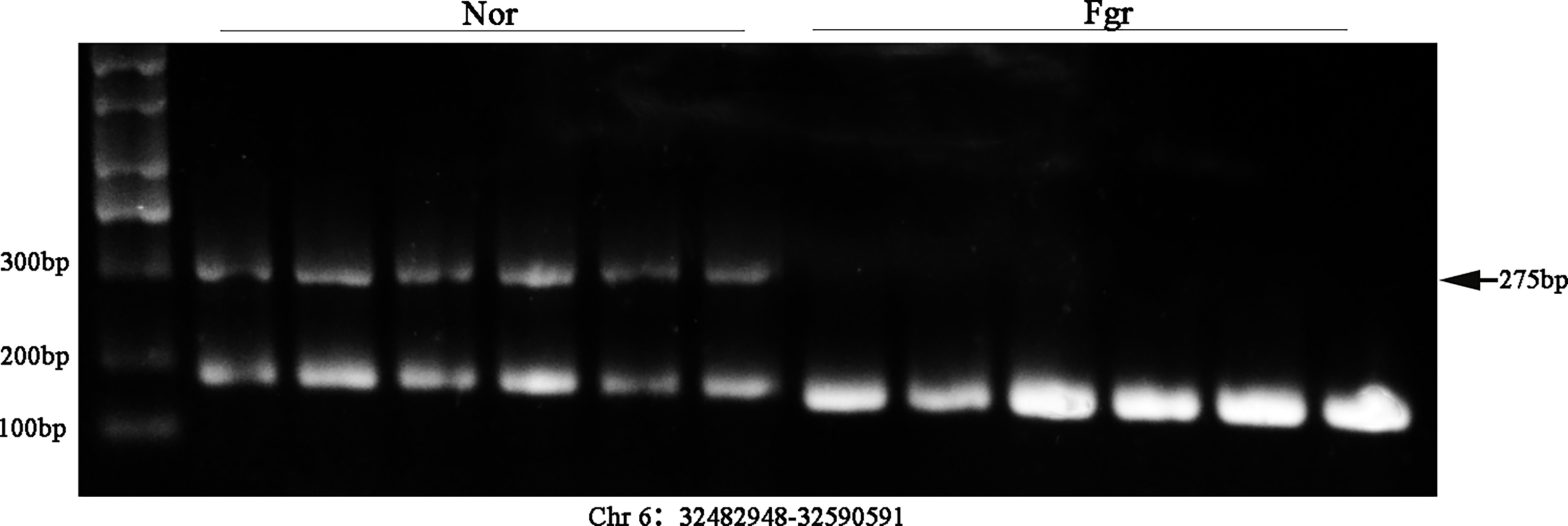
Validation of ecDNA (Chr 6: 32482948-32590591) from 12 placentas (including 6 FGR and 6 normal) using Southern blot.

It has been confirmed that ecDNAs carry complete genes in tumors, which is one reason for promoting disease progression and leading to poor prognosis expression (38). Therefore, we validated whether some ecDNAs were present in 6 normal and 6 FGR placentas (including 3 normal and 3 FGR samples for Circle-seq). After DNA was extracted, PCR amplification was performed by using cross-junction reverse complementary primers (7), e.g. (F: GGGGCCACCTATGTGCAATG R: TGGGAACCAGGAATGAGGGA) (product 275bp) (F: AAGACAGTATGGCGATTCCT R: CATTGTTGGACATTGGGTTG) (product184 bp) (**Supplementary Table 2**), and then further verified by Southern blotting. Encouragingly, we detected the presence of ecDNA (Chr 6: 32482948-32590591) and ecDNA (Chr 8: 71825887-73468945) in placentas (**Figure 7** and **Supplementary Figure 2**), whose variability was consistent with the sequencing results, stimulating our curiosity about the possible role of eccDNAs/ecDNAs in FGR.

## Discussion

This study aimed to determine the origin and characteristics of placental-derived eccDNAs/ecDNAs and further explore their possible effects on pregnancy. We identified that eccDNAs/ecDNAs were abundant in the placenta with significant heterogeneity, and shared the same characteristics in length, GC content, genomic distribution, and repetitive sequence, which similar to previous reports of humans and mice (7, 20, 28). This study is the first report to compare the eccDNAs/ecDNAs in the normal human placenta and FGR human placenta, which is significant for further analysis on pathogeny and mechanism of FGR.

Although we did not observe any difference in the length, GC content (**Figure 3D** and **Supplementary Figure 1D**), genomic distribution, and repetitive sequence between the FGR and normal groups, it was surprising that the amount of eccDNAs/ecDNAs in the FGR placenta was significantly higher than that in the normal group. Abnormal apoptosis of trophoblast cells can cause invasion dysfunction of placental trophoblast cells, and shallow implantation of placental trophoblast cells is a potential pathological mechanism of FGR, thus leading to the occurrence of FGR (39). Given that the production of eccDNAs/ecDNAs is regulated to some extent by apoptosis (40), we speculate that apoptosis plays a critical part in facilitating the DNA elements detached from chromosomes and cyclization in FGR. In addition, placental dysplasia is an important pathological basis of FGR, in which excessive oxidative stress affects the proliferation and apoptosis of trophoblasts (41). The increased level of oxidative stress may aggravate the damage of chromosomes, which will not only promote base oxidation but also lead to single-strand breaks (SSB) and double-strand breaks (DSB) (42). Thereby, they promote the formation of circular elements, which may also be why there are more eccDNAs/ecDNAs in the placenta associated with FGR.

To understand their potential formation mechanisms, we explored the nucleotide modes around the junction locus of the eccDNAs/ecDNAs. Shibata et al. have reported that there are microhomologous base modes around the junction of eccDNAs, and microhomology may promote cyclization of double-terminal DNA through homologous recombination (4, 22). In addition, Sun and his colleagues reported the presence of reverse repeat motif in Arabidopsis (25). In parallel to this, Dennis Lo et al. proposed a new view on the formation of eccDNAs/ecDNAs, where the presence of “double-repeated trinucleotide sequences” on both sides of the junction might be a critical element in the production of those circular elements (14). Repeated deoxyribonucleotide elements usually represent DNA breakpoints and susceptibility sites for R-loop-out events (2, 20, 28). Our statistical analysis found not only direct repeating motifs around the junction of eccDNAs/ecDNAs, but also many reverse repeating motifs, suggesting that microhomology repeats may be the critical factors mediating the formation of eccDNAs/ecDNAs.

The human genome contains many other types of repeated sequences, e.g., TEs, the insertion mutation factor of the eukaryotic genome, affecting the evolution and adaptation of species and individual physiology or disease (43). The proportion of transposons in the human genome is approximately 44% (44, 45). Surprisingly, our results revealed that more than half of the circular entities overlapped TEs in placenta (**Supplementary Table 4**). Furthermore, studies have reported that excessive loss, generation, or insertion of TEs into other DNA sequences on chromosomes of mice during gestation can lead to placental and fetal capillary dysfunction, affecting fetal mice growth and increased embryonic mortality (46, 47). Consequently, a higher percentage of TEs forming circular DNA may also significantly contribute to disease progression.

Additionally, we found that gestational age may also contribute to the formation of these circular entities. Using PCA and Pearson analysis, we discovered that FGR1 deviated from the characteristics of the FGR group. In yeast, the abundance of extrachromosomal rDNA circles (ERCs) is highly related to age (48). Due to the absence of centromeres in circular DNA, including ERCs, eccDNAs/ecDNAs accumulate rapidly in aging yeast through a highly asymmetric mitotic separation process (49). The abundance of eccDNAs in rat lymphocytes and human lung fibroblasts also underwent age-related dynamic procedures (50). However, asymmetric inheritance alone is not enough to promote the accumulation of circular DNA, and the active replication origin carried on circular DNA is an important reason to encourage the production of more copies of circular DNA (49). When elements of circular DNA are neither produced quickly nor have an active origin for replication, they tend to decline with age. Although our findings are very innovative and clinically significant, some limitations still need to be explored further. Since the normal placental tissue matching the gestational week of the FGR group is difficult to obtain, we selected placental tissue delivered at full-term pregnancy as a control. The gestational age might play a role in the differences found. We need more data on placenta with different gestational age to help us explore the effect of gestational weeks for circular elements.

Shibata and colleagues had reported that eccDNAs/ecDNAs originate from the microdeletions of chromosomes (7). Data from the Thousand Genomes Project describe the length of chromosome microdeletions in germline cells peaking at 100bp and 350bp, concentrated in gene regions, with a high frequency of short repeat sequences on both sides microdeletions (51), consistent with our results. Moreover, the origin of eccDNAs is related to the nucleosomes (7), and the characteristics of dinucleosomes are fully reflected in the data of the placenta.

Current studies have demonstrated that eccDNAs/ecDNAs is predominantly derived from genetically dense chromosomal regions. In addition, our data have revealed a genetic preference for the formation of circular DNA, e.g., *PTPRN2* forms 11 unique eccDNAs. *PTRRN2* mediates dysregulation of innate immune pathways. The maternal-fetal interface is in an environment of altered immune homeostasis during pregnancy. Karli S et al. reported that altered methylation of the *PTPRN2* leads to T cell dysfunction and increases reactive oxygen species (ROS) levels, resulting in an increased risk of FGR (52, 53). If so, whether gene cyclization has a similar or opposite effect to methylation makes for fascinating speculation.

To further investigate the possibility of eccDNAs/ecDNAs driving disease progression through its host-genes, we performed a differential analysis between groups. And after GO and KEGG clustering analysis of the host-genes of DEEECs/DEECs, we found an interesting phenomenon: the host-genes of DEEECs mainly cluster in immune-related cellular components, biological processes, and functions. EccDNAs/ecDNAs arise from many functional hotspots, one of which is the recombination of genes encoding T-cell receptor variable regions and immunoglobulin Flight chains (54, 55). Furthermore, the activation of homotypic conversion of B-cell-derived immunoglobulin heavy chains also contributes to the production of eccDNA (56). Studies have demonstrated that altered cytokine patterns at the maternal-fetal interface during pregnancy are an essential mechanism for FGR. Such as increased or decreased populations of dNK cells, macrophages, T cells, dendritic cells, B cells, and NKT cells, which affect placental trophoblast proliferation, apoptosis, vascular endothelial cell activity, etc., thus influencing the pathogenesis of FGR (57–59). We found many host-genes on DEEECs/DEECs have been confirmed to be associated with FGR. Such as the host-gene *KIR2DL1* on eccDNA (*Chr19: 54775137-54839510*), which plays a critical role in the pathogenesis of FGR by binding to HLA-C2, and can increase the secretion of cytokines and thus enhances trophoblast invasion (60, 61). As described above, we conjecture that the imbalance of the maternal-fetal interface immune defenses drives the production of more eccDNAs/ecDNAs, and host-genes of eccDNAs/ecDNAs activation of immune-related pathways and functions exacerbates FGR in turn.

Presently, the exact function of eccDNAs/ecDNAs is not fully understood. Several reports have described its possible role in disease: (1) Unequal replication inheritance of circular DNA leads to heterogeneity of the disease (9, 38, 62). (2) Due to the cyclizing, topological structure change and the sequence rearrange of those elements (e.g., enhancers are connected to the promoter topological structure) result in enhanced gene transcription (38, 63, 64). (3) eccDNAs/ecDNAs can produce miRNA or siRNA, which interact with mRNA to inhibit translation function (13). (4) The cyclization of DNA does not directly affect the expression of the gene itself. It affects the gene expression that circular DNA is inserted into linear chromosomes to form chromosome recombination (including tumor suppressor gene loci, proto-oncogene loci, or other sites) (62, 65). (5) The integration of DNA from different chromosomes or between humans and viruses affects the expression of genes, such as human papillomavirus from cervical cancer (66) and Torque Teno virus gene in pigeon circular DNA genome (67). Here we have specifically explored other potential functions of circular DNA, such as the relationship with ncRNAs. The results show that the annotations of lncRNAs, tRNAs, circRNAs, miRNAs, and snoRNAs partially even completely overlap with these circles, especially circRNAs.

In mammalian genomes, 4%~9% of the sequences that produce transcripts are lncRNAs. lncRNAs are extensively involved in important regulatory processes such as chromosome silencing, genome imprinting, chromatin modification, transcriptional activation, transcriptional interference, and intranuclear transport (68).In addition, numerous studies have reported the critical role of lncRNAs in pregnancy (69–71). Some meaningful data were also found in our research, e.g., lncRNA-*PVT1* overlapped with eccDNA (*Chr8: 128144301-128144456*), which could significantly promote apoptosis and inhibits proliferation, migration, and invasion of trophoblast cells. Additionally, *PVT1* was also proved cross-talking with many miRNAs and genes involved in maintaining the physiological placenta (72, 73). Thus, in addition to a vital oncogene, *PVT1* is also a key regulator in gestational mediating the development of the placenta.

The primary function of tRNAs is to decode mRNA sequences and transport specific amino acids from aminoacyl-tRNA synthetases (AARs) to ribosomes (74). Besides their typical role in protein translation, tRNAs are actively involved in other biology areas, such as primers for retroviral genome reverse transcription (75). Dutta A et al. found that small fragments of tRNA and tRNA halves also can regulate gene expression, protein translation, reverse transcription transposon activity, transgenerational epigenetic changes and responses to environmental stress during pregnancy, which can influence fetal growth and alter maternal immune activation (76). We found that eccDNA overlapped 22 species of tRNAs with high abundance coverage, which make it worthwhile to explore whether the epigenetic modification of tRNAs altered by eccDNAs can influence disease pathogenesis in the future.

Over the past decade, considerable researches have been conducted on ncRNAs in pregnancy-related diseases, of which miRNAs play a central role. Dysregulation of miRNAs from the placenta was proved to influence placental progression and function and these miRNAs to export to the mother and fetus, thus directly affecting maternal and fetal physiology (77–79). Our data showed that many miRNAs (e.g., *miR-191*, *miR-224*, *miR-374a*, *miR-193b*, *miR-365a*, *miR-4287, miR-590, miR-664b*, *miR-3622b*, etc.) overlapped with the genomic loci of ecDNAs. Considering that they can be involved in the onset and progression of FGR by affecting different cellular pathways and functions including oxidative stress, angiogenesis, immunity, inflammation, etc. (80–83), it is reasonable to believe that ecDNAs mediating FGR progression through interaction with miRNAs is also with high potential. Further, circRNAs, as miRNA sponges, can influence the expression of downstream genes (84, 85), which may affect the invasion and apoptosis of trophoblast cells by acting on certain target miRNAs in the human placenta, thus mediating the onset of pregnancy-related diseases (86, 87). Since this study showed some of the ecDNAs almost completely overlapping with circRNAs, we speculate that some circRNAs are not formed by backsplicing of precursor-mRNAs, but by direct transcription of circular DNA.

SnoRNA comes from a conserved family of nuclear-derived RNAs involved in ribosomal subunit maturation and rRNA processing (88), in addition to snoRNA that increases lncRNA stability and maintains subcellular localization through the formation of Sno-lncRNA (89). Mohandas N et al. described that the snoRNA mediated rRNA maturation defects is a distinctive feature of Diamond-Blackfan anemia (DBA), which leads to cell cycle arrest and increased apoptosis by producing nucleolar stress, affecting P53 activity and activation of its downstream targets, and increasing ROS (90). The locus of some ecDNAs in the genome highly overlapped with the small nucleolar RNA H/ACA box (*SNORA*) and small nucleolar RNA C/D/box (*SNORD*), and *U3*, etc. in the snoRNA family. Studies have demonstrated that the SNORA/SNORD family promotes immune imbalance and aging by increasing the immune system’s dendritic cells (DCs) to affect antigen presentation and energy production pathways (91). Our next step is to investigate whether ecDNAs can mediate the progress of FGR by modifying the expression of snoRNAs.

Cell-free DNA (cfDNA) has been shown great promise in the diagnosis, prognosis, and monitoring of many illnesses (e.g., carcinoma) (92, 93), among which cfDNA analysis for examination of trisomy during pregnancy is widely used with high accuracy (94). Recently, Dennis Lo and colleagues characterized maternal plasma containing high amounts of fetal-derived eccDNAs (14). Given the fact that eccDNAs/ecDNAs are more stable than linear DNA, as well as that the large quantity of eccDNAs/ecDNAs was found derived from FGR placentas in our data, and that some genetics preferred to form circular DNA, these findings may help us to develop a new tool for non-invasive prenatal diagnosis that is superior to cfDNA. Besides, the monitoring of eccDNAs in current studies on cancer-related genes can be used as a therapeutic efficacy target (95). Similarly, the tumor-like nature of trophoblasts (plentiful of genes affecting the proliferation, migration, apoptosis, and immune microenvironment) is closely involved in pregnancy-related diseases. Therefore, establishing of eccDNAs/ecDNAs as a biomarker for pregnancy complications has important clinical value.

Considering that most of the discussions above on eccDNAs/ecDNAs function are still not fully confirmed, Liu, X et al. developed a biotechnology-based on CRISPR-C that can facilitate the artificial release of eccDNA from cells (96). We can verify the effect of altered eccDNAs/ecDNAs abundance and types on gene expression and cellular function by performing CRISPR-C intervention at the cellular level in the following study. In view of this, we may venture to imagine whether targeted interventions in circular DNA also have therapeutic potential, a question that certainly deserves further investigation.

**Figure 8:**
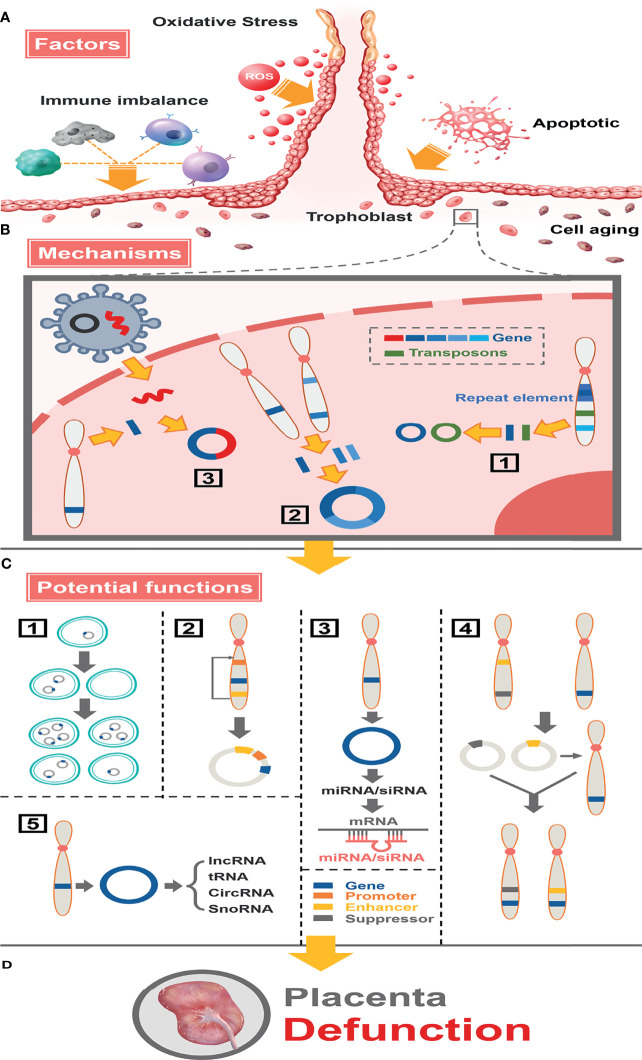
Full landscape of extrachromosomal circular DNA in fetal growth restriction. **(A)** Factors promoting the formation of eccDNAs/ecDNAs in the placenta. Oxidative stress, apoptosis, aging, and immune imbalance at the maternal-fetal interface (such dNK cells, macrophage T cells, B cells, etc.) deteriorate chromosome fragmentation and deletion of gene fragments in trophoblast. **(B)** EccDNAs/ecDNAs of different ontogenetic origins in the placenta and potential mechanisms of formation. (1) Different DNA fragments shed from the same chromosome form multiple cycles by microhomologous recombination. (2) Different DNA fragments shed from different chromosomes are integrated into a cycle by microhomologous recombination.(3) Virus-derived DNA fragments integrate with human chromosomally shed DNA fragments forming cycles. **(C)** Potential functions of eccDNAs/ecDNAs in the placenta. (1) Circular DNA lacks centromere, which inherits genes unequally into offspring cells promoting disease heterogeneity. (2) Effect of altered topology due to cyclization, or promotion of (enhancer linked to promoter topology) gene transcriptional enhancement by sequence rearrangement on the cycle. (3) miRNA or siRNA is produced by eccDNA and interacts with mRNA to suppress gene expression. (4) Cycles carrying enhancers or repressors are chimerized into the genomes of different linear DNAs, forming chromosomal recombination that enhances or represses gene expression. (5) Influence genes expression through network and cross-talk with ncRNAs. **(D)** Placental dysfunction leading to FGR.

In conclusion, we described the landscapes of eccDNAs/ecDNAs in placental tissues of normal and FGR pregnancies by the circle-seq technique and investigated their characteristics and formation mechanisms in detail ***(*** **Figure 8** **)**. Our study also uncovers eccDNAs as a potential driver of FGR through immune signaling pathways. Numerous genomic annotations of ncRNAs overlapped highly with those of eccDNAs/ecDNAs, suggesting the existence of a complex network and underlying biological functions between them. Additionally, the greater stability of eccDNAs/ecDNAs than linear DNA confers excellent biomarker properties, therefore whether their targeted interventions have therapeutic potential is of great benefit for clinical. However, these ideas have high innovation, and the function of these mysterious molecules needs to be further investigated.

## Data Availability Statement

The datasets presented in this study can be found in online repositories. The names of the repository/repositories and accession number(s) can be found below: SRR17054686 and PRJNA784191.

## Ethics Statement

The studies involving human participants were reviewed and approved by The Ethics Committee of the First Affiliated Hospital of Chongqing Medical University. The patients/participants provided their written informed consent to participate in this study. Written informed consent was obtained from the individual(s) for the publication of any potentially identifiable images or data included in this article.

## Author Contributions

HY, JH, and SH designed the research and wrote the manuscript. HY and JH conducted the raw data analysis. HY conducted experiments. HBY supervised part of the study. YT and MC collected the placenta samples. QY performed the statistical analysis. XZ and HQ conceptualized, supervised, and funded this study. All authors contributed to the article and approved the submitted version.

## Funding

This work was supported by The National Natural Science Foundation of China, Grant/Award Numbers: 81771613, 82171679.

## Conflict of Interest

The authors declare that the research was conducted in the absence of any commercial or financial relationships that could be construed as a potential conflict of interest.

## Publisher’s Note

All claims expressed in this article are solely those of the authors and do not necessarily represent those of their affiliated organizations, or those of the publisher, the editors and the reviewers. Any product that may be evaluated in this article, or claim that may be made by its manufacturer, is not guaranteed or endorsed by the publisher.

## Abbreviations

eccDNAs/ecDNAs, Extrachromosomal circular DNAs; FGR, Fetal growth restriction; PCR, Real-time Polymerase Chain Reaction; GO, Gene Ontology; KEGG, Kyoto Encyclopedia of Genes and Genomes; DEEECs/DEECs, Differentially expressed eccDNAs/ecDNAs; ncRNAs, Non-coding RNAs; Nor, Normal; TEs, Transposon elements; CATB, Cetyl trimethyl ammonium bromide; RCA, Rolling circle amplification; PE Paired-end; BWA, Burrows-Wheeler Aligner; TPM, Tags per million; PCA, Principal Component Analysis; SSB, Single-strand breaks; DSB, Double-strand breaks; LTR, Long terminal repeat sequence; ERCs, Extrachromosomal rDNA circles; ROS, Reactive oxygen species; AARs, AminoacyltRNA synthetases; DBA, Diamond-Blackfan anemia; SNORA, Small nucleolar RNA H/ACA box; SNORD, Small nucleolar RNA C/D/box; DCs, Dendritic cells; cfDNA, Cell-free DNA.
