# Supplementary material for: Identification and Characterization of Extrachromosomal Circular DNA in Human Placentas With Fetal Growth Restriction
Source: Front Immunol. 2021 Dec 21;12:780779. doi: 10.3389/fimmu.2021.780779 (PMC8724250; doi:10.3389/fimmu.2021.780779)
Supplement: Supplementary file 1 [file DataSheet_1.docx]

## Supplementary Figures

#
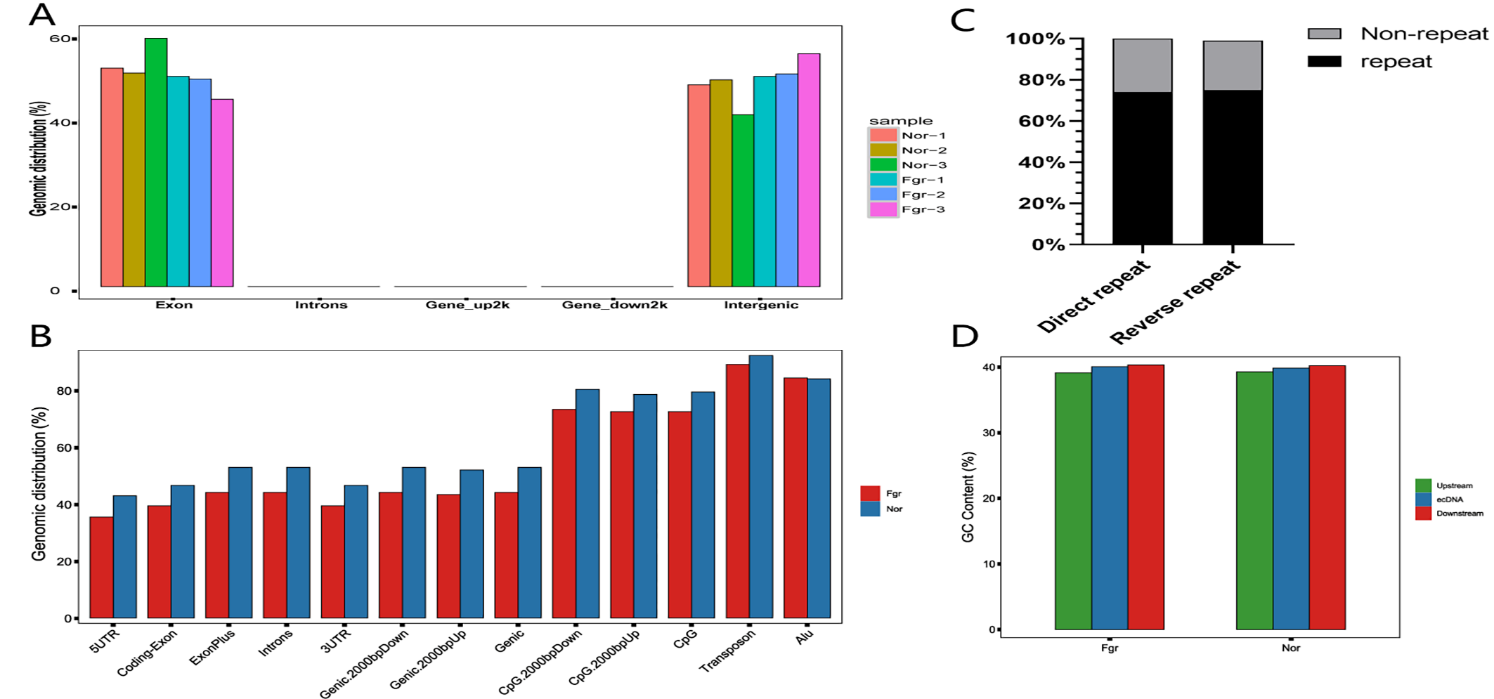


**Supplementary Figure 1. Properties of the loci that release ecDNAs (A)** The [distribution](javascript:;) of ecDNAs of each sample on whole-genome chromosome region. (**B)** Distribution of ecDNAs of FGR and normal groups in the indicated genomic region. (**C)** Percentage of ecDNAs with (black) or without (gray) 4~12bp direct or reverse repeats flanking junction locus at the genomic source. (**D)** Median percent GC content of ecDNAs and the genomic sequences of equal length upstream or downstream of the ecDNAs source loci.

#
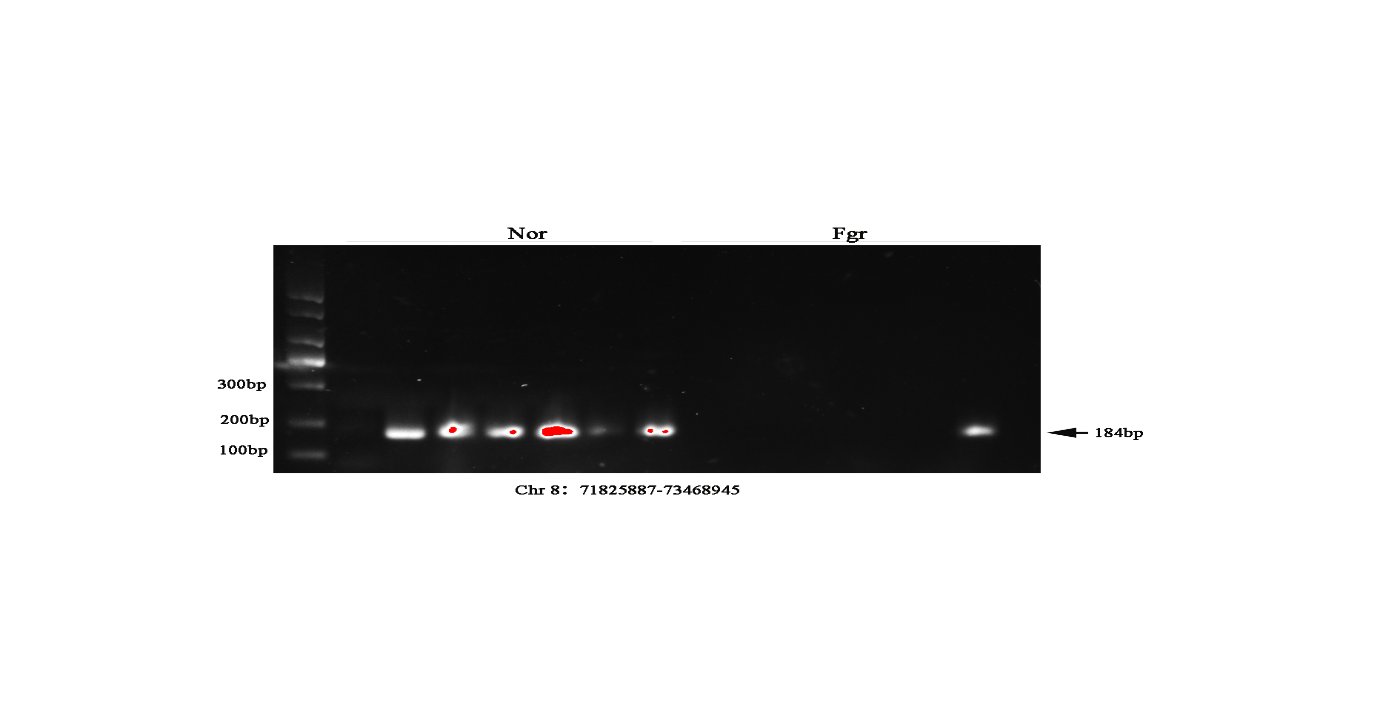


# Supplementary Figure 2. Validation of ecDNA (Chr 8: 71825887-73468945) from 12 placentas (including 6 FGR and 6 normal) using Southern blot.


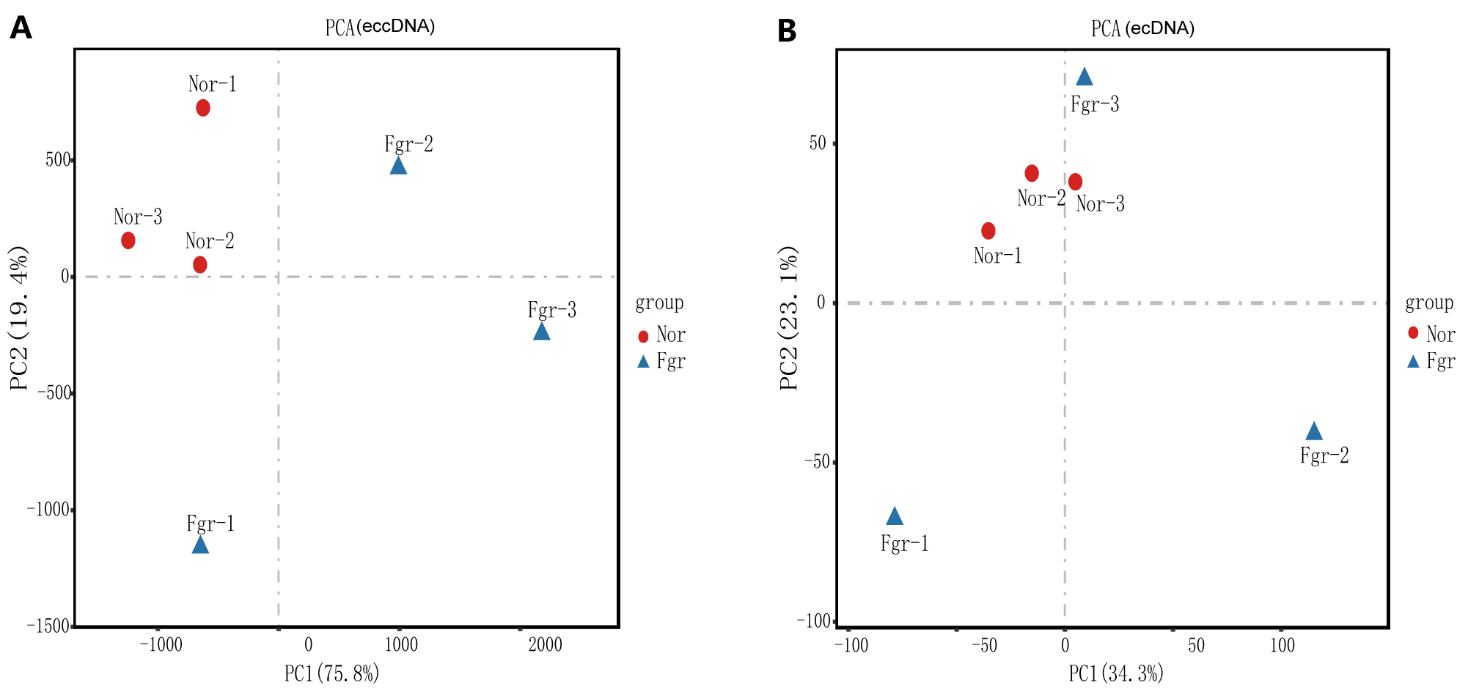


**Supplementary Figure 3. Principal component analysis base on the raw data before normalization. (A)** Principal component analysis base on profile of eccDNAs of six samples. (**B)** Principal component analysis base on profile of ecDNAs of six samples.

## Supplementary tables

**Supplementary Table 1. Maternal age, prepregnancy weight, gestational age,** **neonatal weight, neonatal gender and Apgar score in two groups.**

|  | Maternal age(years) | Prepregnancy weight (Kg) | Gestational age(weeks) | Neonatal weight(g) | Neonatal gender | Apgar score |
| --- | --- | --- | --- | --- | --- | --- |
| Nor1 | 30 | 46 | 39^+6^ | 3181 | boy | 9-10-10 |
| Nor2 | 28 | 45 | 40^+1^ | 3254 | girl | 9-10-10 |
| Nor3 | 28 | 48 | 39^+5^ | 3300 | girl | 10-10-10 |
| Nor4 | 29 | 46 | 39^+6^ | 3181 | girl | 9-10-10 |
| Nor5 | 28 | 49 | 39^+5^ | 3414 | boy | 9-10-10 |
| Nor6 | 29 | 48 | 39^+4^ | 3345 | girl | 10-10-10 |
| Fgr1 | 27 | 47 | 37^+4^ | 2017 | boy | 9-10-10 |
| Fgr2 | 29 | 48 | 34^+4^ | 1618 | girl | 9-10-10 |
| Fgr3 | 30 | 45 | 35^+2^ | 1805 | girl | 8-10-10 |
| Fgr4 | 28 | 49 | 36^+6^ | 2037 | boy | 9-10-10 |
| Fgr5 | 29 | 48 | 35^+3^ | 1718 | boy | 9-10-10 |
| Fgr6 | 29 | 50 | 35^+5^ | 1705 | girl | 8-10-10 |

**Supplementary Table 2. The primers designed on NCBI and PRIMER PREMIER5 software.**

>EccDNA00000118

Cgcctcctcacagttcagcatctcctcacagcacagcacctcctcacagttcagtgtctcaccagtactcagcgcctcctcacagcgcctccccacagcacacagcgcctcctcacagctcagcgcctccccacagcacagcgcctcctcacagttcagcatctcctcacagctcagcacctcacagctcagtgcctcctcacagctcagcgcctcctcacagctcagcgcctcctcacagctcagcgcctcctcacagttcagcatctcctcacagcacagcacctcctcacagttcagtgtctcaccagtactcagcgcctcctcacagcgcctccccacagca

**Primer pair 1**

|  | **Sequence (5'->3')** | **Template strand** | **Length** | **Start** | **Stop** | **Tm** | **GC%** |
| --- | --- | --- | --- | --- | --- | --- | --- |
| **Forward primer** | CACAGTTCAGTGTCTCACCAGTA | Plus | 23 | 45 | 67 | 59.93 | 47.83 |
| **Reverse primer** | CTGAGCTGTGAGGTGCTGAG | Minus | 20 | 192 | 173 | 60.39 | 60.00 |
| **Product length** | 148 |  |  |  |  |  |  |

**Primer pair 2**

|  | **Sequence (5'->3')** | **Template strand** | **Length** | **Start** | **Stop** | **Tm** | **GC%** |
| --- | --- | --- | --- | --- | --- | --- | --- |
| **Forward primer** | CAGTTCAGTGTCTCACCAGT | Plus | 20 | 47 | 66 | 57.10 | 50.00 |
| **Reverse primer** | GGCACTGAGCTGTGAGGT | Minus | 18 | 196 | 179 | 59.25 | 61.11 |
| **Product length** | 150 |  |  |  |  |  |  |

>EccDNA00000506

ATAATGGGTCATGCAGGAACTTGTAGGCAAGTTCCTAGTAATTGGTGAAGCTTCAACTATAGACGAAAATTTGTCTTCCTCGTTTCTTTCCTGGCACAAGAATGTGAGGAAGCAGAACCACAGATAATAAAGAAAGAGGAGCCCTGGTGACAGCGAGGTGCTGGCGAGGACGGAGACCACTGAGCAGATGAGGAAGCCCCGGAGGGAGACCACTGAGCAGATGAGGAAGCCCCGCCCTCCCTGCACCTGCTCCTGACCGGGCCTCCTGCTCTGTGGGCCCCGCGCGCCCCCTGCTGGCGCTGAGCAGCACCTGCGCCGGTCCCCTCCGCCTCCCTGCACGGAGGTTTTTGTCTGGGCTCACACTCACCTCCCCTCACTGTGTCTCTCGCACAGTAATACACGGCCGTGTCCGCAGCGGTCACAGAGCTCAGCTTCAGGGAGAACTGGTTCTTGGACGTGTCTACTGATATGGTGACTCGACTCTTGAGGGAGGGGTTGTAGTTGGTGCTCCCACTGTAATAGATATACCCAATCCACTCCAGTCCCTTCCCTGGGGGCTGCCGGATCCAGCTCCAGTAGTAACTACTGATGGAGCCACCAGAGACAGTGCAGGTGAGGGACAGGGTCTCCGAAGGCTTCACCAGTCCTGGGCCCGACTCCTGCAGCTGCACCTGGGACAGGACCCCTGTGAACAGAGAGACCCACAGTGAGCCCTGGGATCAGAGGCACCTCCCATATCCCCATGTCTGGATCCCTGAGATACTCACATCTGGGAGCTGCCACCAGGAGAAGGAAGAACCACAGATGTTTCATGTTCTTGCACA

3’:aaaattctgcagtctgaatgttgaagattagtgtgctggcagggctggttcttggcgcggcttcttcctggcttgcactgggccaccttctagtgcactatgtctccacatggcctcttctctgcctgcacgtgaaaagtgagaggtctctggtgtctcttcctcttcttataaagacaacacgtctattgcattagggtcttacaattatgaccacatttaaccttaattatttcattaaaattccaatatagatccattgaatttaaggtttcatcatatgaatttcaaagagggtacaattcagttgatgataCAAATCAAGAGATGGTGAGAAGCATTAGAATATATATTTTTTAATCGATAAGTTATAATGGGTCATGCAGGAACTTGTAGGCAAGTTCCTAGTAATTGGTGAAGCTTCAACTATAGACGAAAATTTGTCTTCCTCGTTTCTTTCCTGGCACAAGAATGTGAGGAAGCAGAACCACAGATAATAAAGAAAGAGGAGCCCTGGTGACAGCGAGGTGCTGGCGAGGACGGAGACCACTGAGCAGATGAGGAAGCCCC

**Primer pair 1**

|  | **Sequence (5'->3')** | **Template strand** | **Length** | **Start** | **Stop** | **Tm** | **GC%** |
| --- | --- | --- | --- | --- | --- | --- | --- |
| **Forward primer** | TGGGTCATGCAGGAACTTGTA | Plus | 21 | 5 | 25 | 59.30 | 47.62 |
| **Reverse primer** | TGTGAGCCCAGACAAAAACCT | Minus | 21 | 362 | 342 | 60.06 | 47.62 |
| **Product length** | 358 |  |  |  |  |  |  |

**Primer pair 2**

|  | **Sequence (5'->3')** | **Template strand** | **Length** | **Start** | **Stop** | **Tm** | **GC%** |
| --- | --- | --- | --- | --- | --- | --- | --- |
| **Forward primer** | AGGCAAGTTCCTAGTAATTGGTGAA | Plus | 25 | 25 | 49 | 60.22 | 40.00 |
| **Reverse primer** | TACTGTGCGAGAGACACAGTGAG | Minus | 23 | 395 | 373 | 61.90 | 52.17 |
| **Product length** | 371 |  |  |  |  |  |  |

>EccDNA00000650

agtttaactcacagagctgaacattcctttggatggagcagtttcgaaacacactatttgtagaatgtgcaactggatatttgggcctccctgaggatttcgttggaaacgggataaaccacacagaactaaacagaagcattctcagaacattcttcgtgatgtttgcattcaactcacagtgttgaacctttctgtgatagttcaggttggaaacggtctttctgtagaaactgcaagtagatatttggacctctctgaggatttcgttggaaacgggataaaccgcacagaactaaaacagaagcattcacagaaaactcttggtgttgactgagtttaactcacagagctgaacattcctttggatggagcagtttcgaaacacactatttgtagaatgtgcaactggatatttgggcctccctgaggatttcgttggaaacgggataaaccacacagaactaaacagaagcattctcagaa

F: AACATTCCTTTGGATGGAGCAG

R:TGTGCGGTTTATCCCGTTTC

length:273

>EccDNA00000906

5’:GAGACAGAGAAAGAGAGCATTAGGCCATAGAGCAGGGGAGTGAGTTCTCAGGTCAGGTGTGAGGGGAGCTGTGACAAGGAAGATCCCCCCTGAGGAAACTGCCCCTTCTCCTTCCAGGTCTATATGAGAAACCTTCTCTCTCAGCCCAGCCGGGCCCCACGGTTCAGGCAGGAGAGAATGTGACCTTGTCCTGCAGCTCCTGAGGAAACTGCCTCTTCTCCTTCCAGGTCTATATGAGAAACCTTCTCTCTCAGCCCAGCTGGGCCCCACGGTTCTGGCAGGAGAGAATGTGACCTTGTCCTGCAGCTCCCGGAGCTCCTATGACATGTACCATCTATCCAGGGAAGGGGAGGCCCATGAACGTAGGCTCCCTGCAGGGCCCAAGGTCAACGGAACATTCCAGGCTGACTTTCCTCTGGGCCCTGCCACCCACGGAGGGACCTACAGATGCTTCGGCTCTTTCCATGACTCTCCATACGAGTGGTCAAAGTCAAGTGACCCACTGCTTGTTTCTGTCACAGGTGAGGAAAGCCCATGGCTGTCCCATGTCCTATGATCCTAGAGCCTTAGCTGAGGAGCTTCCTGCTGAGGATGGAGAGAAGCATGGACAGATGCAGAGAGAAGACGCAGCCTCGGTGTGAGGGAGGGATCAGGGCACAGGATGGCCGACAGGGCACCTCCAAACCCTCCTACATG3’:GATGATTGACGGATAGACAATTGATAGATAAATAGATGATATATAGATATAGATGACAGGTAGAGAATTTGTAGATAGGCACCGAATAGATAAATAGATGGATTGATAGATAATAGATAGAAATATGCAGAAAGTTATGAACGGGACACAAACTGAGAAACTCAGAGTTAAAAAAAGTAACATCAAGTCAACCAATCCAAGGAGAGCCAGAGAGAATAAAACAATCCAAAAACGGAAAACATAACTAGAGGTAGGGAAGTGAGGTCAGAGACCTACAGAGACAGAGAAGGTGGAAGGAGGAAATAGACATGAAGAGAGATAGGGTGGAGGGTGAGACAGAGAAAGAGAGCATTAGGCCATAGAGCAGGGGAGTGAGTTCTCAGGTCAGGTGTGAGGGGAGCTGTGACAAGGAAGATCCCCCCTGAGGAAACTGCCCCTTCTCCTTCCAGGTCTATATGAGAAACCTTCTCTCTCAGCCCAGCCGGGCCCCACGGTTCAGGCAGGAGAGAATGTGACCTTGTCCTGCAGCTCC

**Primer pair 1**

|  | **Sequence (5'->3')** | **Template strand** | **Length** | **Start** | **Stop** | **Tm** | **GC%** |
| --- | --- | --- | --- | --- | --- | --- | --- |
| **Forward primer** | CATTAGGCCATAGAGCAGGGG | Plus | 21 | 18 | 38 | 60.00 | 57.14 |
| **Reverse primer** | CCTCCCCTTCCCTGGATAGAT | Minus | 21 | 353 | 333 | 59.85 | 57.14 |
| **Product length** | 336 |  |  |  |  |  |  |

**Primer pair 2**

|  | **Sequence (5'->3')** | **Template strand** | **Length** | **Start** | **Stop** | **Tm** | **GC%** |
| --- | --- | --- | --- | --- | --- | --- | --- |
| **Forward primer** | GTTCTCAGGTCAGGTGTGAGG | Plus | 21 | 44 | 64 | 60.00 | 57.14 |
| **Reverse primer** | TGCAGGGAGCCTACGTTCAT | Minus | 20 | 376 | 357 | 61.26 | 55.00 |
| **Product length** | 333 |  |  |  |  |  |  |

**Primer pair 3**

|  | **Sequence (5'->3')** | **Template strand** | **Length** | **Start** | **Stop** | **Tm** | **GC%** |
| --- | --- | --- | --- | --- | --- | --- | --- |
| **Forward primer** | AGCTGTGACAAGGAAGATCCC | Plus | 21 | 67 | 87 | 59.72 | 52.38 |
| **Reverse primer** | AGCCTACGTTCATGGGCCTC | Minus | 20 | 369 | 350 | 61.97 | 60.00 |
| **Product length** | 303 |  |  |  |  |  |  |

>EccDNA00000983

aatcaTCGAGTGGACACGAATGTAACCATCACTGAATTGAATCGAATAGAATCATCAAATGGAATTGAACGGAAACAACATCGAATGGAATCGAATTTAGTCATTGAATTGCATTGAGTGGAATCATCATTGAATGGAATCTAAGGAAATCATCGAATGGACTCGAGTGGAATCATCGAATGGACTCGAGGGGAATCATCatcatcGAATGGACTCGAGTGGAATCATCGAATGGACTCGAGGGGAATCATCATTGAATGGAATCGACAGTAATCAACGAATGGACTCGAACAGAATCATCATCGAATGGAATCGAATGGAATCAAAGAATGGACTCTAATGGAGTCATCATCTAATGGAATCTAATGGGATCATCTAATGGACCTGAAAGGAATCATCATTgaatggaatcgaatggaatcatcgaatggactcgaatggaatcatcatcgaatggaatcTAATGGAATCATTGAATGGACTCGAATGGAATAATCGAATGGGCTTGAGTGGAATCATCATCAAAtggaatcgaatggaatcatcaaatggactcgaatggaatcatcgtcaaatggaatcgaatggaaTCATCATCAAATGGAATCGAAGGGAATCCTCATCGAATGGAATCGAACGGAATCGTCATCGAATGGTATCACCAAATTGAATCGAATGGAATGATCGTCAAAGACAATCGAATTGAAACATCGAACGGGATTGAACGGAGTCATCGAATGGAATCGATAGGAAACATCGAAAGGATTCAAATGGACTCATCATCGAATGGAATCAAATGGAGTCATCCAATGGACTCGaatggaatcatcatgaaatgggatcattatcaaatggaatcGAATGGAGTCATCGAATGGACACGAATGAATGAACAAATGGACTCGAATGGAAACATCAAATAGAATCGAATGGAATCATCAAAAGGAATTGAATGGAATTATTGAATGGACTCGAATGGAATCATCTAATGGACACGAATGGAATAATCATAAAATGCAATCGAATGGAATCATCAaatggactcgaatgaatggaatcattaaatggactcgaatggaatcatcgaatggactcaaatggaatcatcatcaaatggaatcgaatggaatcatcgaatggactcgaa3’:ATGTAATTCAATCGAATGGACATGAAtggaatcatcattgaatggaatcaaatggaatcctcatcgaatggaatcgaatggaatcatcAAATGGAATAGAATGGAGTCATCGTCGAATGTAATCGAATGTAATCATCGAATGGCATCGAATGGAATCGTCGACTGGAAAAGAATGGAATCATCATCGAATGGAAATGAATAGATTCACAGAATGAAATcgaatggaatcatcatcgaatggagtctaatggaataatcatcgaatggaatagaatggaatcaTCGAGTGGACACGAATGTAACCATCACTGAATTGAATCGAATAGAATCATCAAATGGAATTGAACGGAAACAACATCGAATGGAATCGAATTTAGTCATTGAATTGCATTGAGTGGAATCATCATTGAATGGAATCTAAGGAAATCATCGAATGGACTCGAGTGGAATCATCGAATGGACTCGAGGGGAATCATC

F: CACGAATGTAACCATCACTG

R: ATGATGATTCCTTTCAGGTC

LENGTH:387

>EccDNA00001057

acagtgtgtgggcagtgcagtgtgtgcatacaatgcagcatgtgtccagtgcagtgtgtgtagtgcagtgtgtctatagtacagtgtgtgtgcagtgcagtgtgtgtacagaacggtgtgtgtgcagtgcggtgtgtgtaagtacagtgtttacagtacagtgtgtgtgaagtgcagtgtgtatgctgtacagtgtgtgtagagtacagtgtctgtgcagtgcagcgtgtgtgcagtgcagtgtgtgtacagaacagtgtgtgtgcagtgcagtgtatgtactatgcagtgtgtgtactgtgcagtgtgtgtactgtgcagtgtgtgtgcagtgcagtgtgtgtgcagtgcagtgtgtgtactatacagcatgtgtgttgtgcagtatgggtatagtgctgtgtgtgctgtgcaggatgggtatagtgcagtgtgtgtactgtgcagtgtgtgtactgtacagtgtgtgtgtacagtacagtgtgtgggcagtgcagtgtgtgcatacaatgcagcatgtgtccagtgcagtgtgtgtagtgcagtgtgtctatagtacagtgtgtgtgcagtgcagtgtgtgtacagaacggtgtgtgtgcagtgcggtgtgtgtaagtacagtgtttacagtacagtgtgtgtgaagtgcagtgtgtatgctgtacagtgtgtgt

F: TGCATACAATGCAGCATGTG

R: ATACCCATCCTGCACAGCAC

LENGTH:400

>EccDNA00001231

taacagttcttgctctctcaggaatggattcattctcaccagattggttgttataaagccaggatgattccaggtttgatccatcttcacacgtgtctggtgcccatttgactttctccaccatgttttgatgcaactcaaaagcccttactagaccaggagcagtggctcatgcctgtaatcccagcactttgggaggccgaggtgaatcacctgagatcaggagttggagaccagcctggccaacatggtgaaaccccgtctctacaaaaatacaaaaattagttgggggtggtggtgggcgcctgtaatcccagctactcaggaggctgaggcaggagaatgccttgaacccaggaagcagaggttgcagtgagctgggatcacgccactgcactccaacctgggcaagagagcaagactccatctcaaaaaaGATACTCAATAAATAAATAAATAACAGGGAGGGATTGGTGGTATATCTGGGgctatggtttggatgtggcgtgtttgtctccaacaaatttcatgttgaaattggatccccagtgaggtagtgtggggaggtggggccttgtgggaggtgtttggatcatggagacagatataacagttcttgctctctcaggaatggattcattctcaccagattggttgttataaagccaggatgattccaggtttgatccatcttcacacgtgtctggtgcccatttgactttctccaccatgttttgatgcaactcaaaagcccttactagaccaggagcagtggctcatgcctgtaatcccagcactttgggaggc

F: GCCCATTTGACTTTCTCCAC

R: TATACCACCAATCCCTCCCT

length:380

>EccDNA00001573

aaagtgctgggattacgggcatgagccaccgcacctggcccagtttagtgttttttttgttttgttttgtttttgagatggagtttcgctcttgttgcccaggctggagtgcaatggcacgattttggctcaccgcaacctccgcctcccaggttacagcgattctcctgcctcagtttcccaagtagctgggattacagacacgcaccaccgcacctggctaatttttgtattttcagtacagatgggatttcaccatgttggccaggctggtctcaaacgcctgacctcaagtgatccatccaccttggcctcccaaagtgctgggattacaggtgtgagctactgcgcccagccTCATCTACGTTTTTGTTGCAGCAAACACTACAATTATACTTGCGTATGCCATCTTAATTCTTAAAACCTGTCTTATCTTTTTTCTGATGCCATTTGCTGCCTCTGGTCTTCTAATCATATTGCTTGCTACTAGCCCTGTTTCAGAGACTAAAAAATAACCCTAAATGGTATAACTTAACAACCTTACTGGTTGGTATTAAGTGGTTTTAAAAATTCACCTCCTGCCTCTTACTGAACCAAGTTCAGTGTGTTGAATTAGTGAGTAATTCCATCCTCGGCAGCTATAACAACATGCAGTTTCTGTTTCCCAGATCTTTTTTATGCTTCATTAAAAATCACTATTTTgccaggcacagtggctcacgcctgtaatcccagcactttgggaggccgaggcaggcagatcctgaggtcaggagattgagaccatcctggctaacacggtgaaaccccatctctactaaaaatacaaaaaattagccgggcgtggtggcaggcgcctgtagtcccagcttctcgggaggctggggcaggagaatggcgtgaacccaggaggcggagcttgcagtgagccgagatcgtgccattgcactccagcctgggcaacagagcaagactccatctcaaaaaagaaaaaaaaaaaagaaaaaTCACTATTTTGTGTGTGTGTGACagtcttgctctgttgctcaggctggagtgcaatggcacgatctcggctcgctgcaacctccgcctcctaggttcaagcagttctgcctcggccttctgagtagctgggattacaggtgcccaccaccatgccagcgaattttttgtatttttagtagagaaggggcttcaccatgttggccaggctggtctcgaactcctgacctcaggtgatccacctgcttcagcctcccaaagtgctgggattacaggcatgagccaccacacccagccAAAAATCACTTTCTTAAGATGTTTTATTTACACAAAATAAATAGGAAGATTTCTTggcttggtgtggtggctcacccctgtaatctcagcactttgggaggccaagacaagaggattgcttgagcccaggagttcatgaccagcccaggcaacatagcaagacccccgtctctacaaaaaatttaaaaattggctgggcatTAAAAGTTTTAAtttttttttttCTCCTCCATAAATTAGGAATAACTTACTTTGAAACATAGGAATAAACTTAACTTGAATTACTGGTATTTGTATTTGAAAAACTAAAGAACTGCGGCATCAATTATATGGACAGACAGCAGTTTATGTCTCTTCTGAGCAGCGTACATGGTGGCCACATAGAGCAGCCCAAGGAGCCCCTGGCTAACGCAGCCAGCAGCAGGCACAAGTGGTAGTTAGCTGTATGCTGTCCACTGCAGGACATCTTCAGATCAATCCTTTTCAGAGTCTATTACGAACTCACAGTATCTTACTCGTAGAAGAAATCTGGCCATAGGTATTTGTCTGACTTGGCCTAACCTATAATTCACTCAAAGTTCAGtttagtttagtttttttttgtttgttttttgttttttgtttttttttgagacggagtttcactcttgttgcccaggctggagtgcaatggcgcaatcttggctcactgcaacttctgcctcccaggttaaagcgattctcctgcctcagcctcccaagtagctgggattacaggcatgtgccaccacgccaggctaattttgtatttttagtagagacagagtttctccatgttggtcaggctggtctcgaactcctgacctcaggtgatctgcccacctccgcctcccaaagcgctgggattacaggcgtgagccaccacgcccagcttagttttgatttttgttttttggtttttttggagacagagtcttgctctgtcactcaggctggagtgcagtggcaggatctcagctcattgcaacctctgcctcccaggttcaagtgattcttctgcctcagcctcccaagtagctgggattacaggtgctctccaccatacctggctaatttttttatgtatttttagtagagacggggattcaccatgttggccagtctggtctcgaactcctgaccttaggtgatccacctgcctccaccttccaaagtgctgggattacgggcatgagccaccgcacctggcccagtttagtgttttttttgttttgttttgtttttgagatggagtttcgctcttgttgcccaggctggagtgcaatggcacgattttggctcaccgcaacctccgcctcccaggttacagcgattctcctgcctcagtttcccaagtagctgggattacag

F: CCAGGTTACAGCGATTCTCC

R: TCACTTGAGGTCAGGCGTTT

LENGTH:149

>EcDNA00000033

5’cagaggaattcccagtaacttccttgtgttgtgtgcattcaactcacagatttgaatgattctttccacagagcagatttgagacactcttttgttagaatttgtaagtggagaaatcagcagctttgaggtcaatggtagaaaaggaaatatcttcgtataaaaactagacagaatgattctcagaaactcctttgtgatgtgtgcgtccaactcgcagagtttaacctttcttttcatagagcagttaggaaacactctgtttgtgaagtctgccagtggatattcgcacctatttaaccttcgttggaaacgggatttcttcatattatgctagacagaagatttctcagtaactactttgtgttgtgtgtatgcaactcacagacttcaaccttattttagacagagcagatttgaaacactctttttgtgcaatttgcaagtggagatttcaagcgctttgatgccaatggtagaaaaggatatatcttcgtataaaaacaagacaaaatctttcccagaatctgtgtagtgatctgtttgtttaactcacagagtttaacctttcttatcatacagcattctggaaaccctcagtttgtaaagtctgcaagtggatatttggacctcttagatgccttcgttggaaacggtttttcttcatataatgctagagggaagaattcttaataacttctttgtgttgtgtgtattcaactgacagagttgaaccttcctttagacagagcagatttgaaagtctctttttgtggaatttgcaagtggagatttcaagcgctttgaggccaaaagcagaaaaggaaatattttcctataaaaactagacagaatcattctcagaaactgctctgtgatgtgtgtgttcaactcacagagtttaactttcttttcattcagcagtttggaaacactctgtttggaaagtctgcacgtggatattttgacctctttgaggccttcgttggaaacgggtttttttcatgtaaggctagacagaagaaatctcagtaacttccttgtgttgtgtgtattcaactgacagagttgaaccttcctttagacagagcagattcgaaacactctttttctgcaatttgcaagtggagacttcaagtgctttgaggccaaaggcagaaaaggaaatatcttcgtataaaaacccgacagaatcattctcagaaactgctctgtgatgtgtgcgttcaactcacagagtttaacttttcttttcattcagcagtttggaaacactctgtttgtaaagtctgcaagtggatatcttggcctcttagaggccttc

3’:tttcttttcaaagagcagttaggaaacactctgtttgtaaagtctgcaagtggatattcagacctctttgaggccttcgttggaaacgggatttcttcatattatgctagacagaggaattcccagtaacttccttgtgttgtgtgcattcaactcacagatttgaatgattctttccacagagcagatttgagacactcttttgttagaatttgtaagtggagaaatcagcagctttgaggtcaatggtagaaaaggaaatatcttcgtataaaaactagacagaatgattctcagaaactcctttgtgatgtgtgcgtccaactcgcagagtttaacctttcttttcatagagcagttaggaaacactctgtttgtgaagtctgccagtggatattcgcacctatttaa

F:TTCCCAGTAACTTCCTTGTG

R: TTTCTACCATTGGCATCAAA

LENGTH:：474

F: CTGTTTGTGAAGTCTGCCAGTG

R: GAGGGTTTCCAGAATGCTGTATGA

LENGTH:：339

>EcDNA00000190

5’:cacaatgagataccaccacacaccagttagaatggcaatcattaaaaagtcaggaaacgacaggtgctggagaggatgtggagaaataggaacacttttacaccgttggtgggactgtaaactagttcaaccattgtggaagacagtatggcgattcctcagggatctagaactagaaataacatttgacccagccatccattattgggcgtatacccaaaggattataaatcatgctgctataaagacacatgcacacgtatgtttattgtggcactattcacaatagcaaagacttggaaccaacccaatgtccaacaatgttatactggattaagaaaatgtggcacatatacaccatggaatactatgcagccataaaaaaggatgagttcatgtcctttgtagggacatggatgaagctgcaaaccatcattctcagcaaactgtctcaaggagaaaaaaccaaacaccacatgttctcactcatagatgggaactgaacaatgagaacacatggacacaggtaggggagcgtcacacactgaggcctgttgtggggtgggaggaggggggagggatagcattaggagatatacctaatgttaaatgacgagttaatgggtgcagcacaccaacatggcacatgtatacatatgtaacaaacctgcacgttgtgcacatgtaccctaaaacttaaagtataatttaaaaaaTATATTGTCTTTGTTTGTGTTTCCATCAATAATAGCATAATTTGGCTTGAGTGAAGAGTGAAGGTGGAGAAGGTGAGCAGAGCACGATACTGTTTTGCCTATTCTCATCTTAAAACACAGATTTGAATCATTACACGTGTGAAATGCAGGCTTTCCATGGGAAGAAGTAGAATGGTTCTTGTATTTCTGGAAAGACACAGCAAGTGAATCTGCTACTTTATAGCttttttttttttttttttttttgagaccgagtctcaccctatcgtccaggctggagaacaatggggcgatcttggctcactgcaagcccctccacccaggttcaaacgattctcctgcctcagcctctcgagtggctgggattcaggcacccaccaccaggctccactcatttttgtatttttagtagaaatgtggtttcaccatattggccaggctggcctcgaactcctgacctcgtgatccacctgcctcggcctcccaaagtgctgggattacaagcgtg

3’:cataggcataggcaaggacttcatgtctaaaacaccaaaagcgatggcaacaaaagccaaaattgacaaatgggatctaattaaactaaagagcttctgcacagcaaaagaaactaccatcagagtgaacaggcaacctacagaatgggagaaaatttttgcaatctactcatctgacaaagggctaatatccagaatctacaaagaactcaaacaaatttacaagaaaaaacaaataaccccatcaaaaagtgggcgaaggatatgaacagacacttctcaaaagaagacatttatgcagccaacagacatatgaaaaaatgctcatcatcactggccatcagagaaatgcaaatcaaaaccacaatgagataccaccacacaccagttagaatggcaatcattaaaaagtcaggaaacgacaggtgctggagaggatgtggagaaataggaacacttttacaccgttggtgggactgtaaactagttcaaccattgtggaagacagtatggcgattcctcagggatctagaactagaaataacatttgacccagccatcc

F: AAGACAGTATGGCGATTCCT

R: CATTGTTGGACATTGGGTTG

LENGTH:184

>EcDNA00000163

5’:GTTGACCGGAGAGCTTAATCTGTACCTATTTACAGGTAGAGATGTCTGTAATAAGTTAAAGGAAATTGAAAGTTAGTTAATAATTTAATCTGAGTAAAAAGAGTTTTTTCAAGTGTGTCTCCTGATGCTGCCCCCAAGTTTAGTGTCATCTCCAGAACACACACAGGCAAGGGGCTTGCAGGGGCCACCTATGTGCAATGCCCAAGTTTagtggcacctccagaacacacacaggaaggggcttgcagggaccacctatgtgcaatggagggtctgaaggtgcctttgtatagcacttaccctaacaatgtgataaggtcaactgtgcaatcgaagtattcaggggtctgagagattGATCAAGGACTCAAAGTCAGCTGTTGACAGAAAAACACTGCTGTAAAATAATTAATATTTTATGTGAAGAGTGTTCAATCCCTCATTCCTGGTTCCCATTAGGATTTCCTCATTTGATTGAGGTTATGGCCCTTTACTATTATGCTTCTTTTGATTTATCATAAGGGAAGATATAAGAAGACTGTGCTAACTAATACGTTACAGAATGTTCAGGAAAGAGAACCCTAGGGAAAAACTATGAATTACATCAGCTGATGTAATCATGTAATTTTAAACATATAATTCTACATTTAGATAATTATTATGCTTTATATTAATATAAATGTGACATCTAAGATTCAGAATGGACTTCAAAGTACAACTATACATATAAAGCTCTGCATTAATTCACACTGTACCACAGTTGAGATAGGCACTCCTTCCTTAT

3’:CTTCTGAAACAGAATTTCCTTCTTTAAAGGATTGTTTTAATTTAGCACTTGGAAGGTTTGAACCAGTTGCATGTAAAACACCTTAATTGGGGAGCTATTGTAGCCAGCTCTGTGCTGGTCAGTGATGTGTTCACAAGTTTGAGCCTTGTAAGAGCATTCATTTCCCACTTGACAAGACAACTATTTGCAGGAGTGAGTGTGAGTGTGTTTAGGAGTAAAGGAGATGgagggaacatggccgcaaatcaggagacctttaatctggtccttattgcaccatatcttaatgttgtagatttgggaaaattacgtcatgtctcacagttgaaatgaaggcaccatgatctttcaggtctttcaacactagaaaatgtgattctgtgGATGCCTCAAGGAGCAGCAGCTCCGGGTATCTGATGATATGACAGAATGACAGCTGTTGACCGGAGAGCTTAATCTGTACCTATTTACAGGTAGAGATGTCTGTAATAAGTTAAAGGAAATTGAAAGTTAGTTAATAATTTAATCTGAGTAAAAAGAGTTTTTTCAAGTGTGTCTCCTGATGCTGCCCCCAAGTTTAGTGTCATCTCCAGAACACACACAGGCAAGGGGCTTGCAGGGGCCACCTATGTGCAATG

F: GGGGCCACCTATGTGCAATG

R: TGGGAACCAGGAATGAGGGA

LENGTH:275

>EcDNA00000123

5’:ctgaagtgacagtgttgaacctatcttttgattcagcagttttgagtctctctttttacagaatctgagagtggatatttggagcgctttgaggcgcactgtggaaaatgaaatatcttcacacaaaaactacacagaagcattctgagaaacatctttgtgaggtgtgcactgaagtcacattgttgaacctatgtttttctctttttacagaatctgagagtggatatttggagcgctttgaggcgtactgtggaaaatgaaatatcttcacacaaaaactacacagaagcattctgagaaacttctttgtgatgtgtgcattcatctcacagagttgaatgtctctgttgattgagcagttttgaaacactctttttgtggaatctgcaagtggatatttggagctcattggggcctactgtggaaaatcaaatatcttcacataaaaactacacagaagcattctgaaaaacttctttgtgatctgtgcattcatctcacagagttggatgtttctattgatatagcagttttgaaacactcttttttagaatctgtaaatggatatttggagccttttcaggcctacagtgtagaaggaaatatcttcacataaaaactatgcagaagcattctgagaaactactttgtgatgcgtgcattcatctcacagggtacaacctttctttggattgagcagttttgaaacactctttttgtagaatctgcaagtggatatttagagtgatttgaggcctattgtggaaagggaaatttcttcaactaaaaactacccagaagcattctgtgaaacttatttgcgatgtgtgcattcaactcacattgttgaaggtatctgttgattgagtagtttagaatctctctttttgtagaatctgcaagtgaatatttggggccctattttgccctatattggaaaaggaaatatcttcaaatagaaactacacagaagcattcagagaaccttctttctgatgagtgcattcatcacagagttgaacctttgttttgatttagcagttttgacacaatctttccgtacaatctggaagtgaatatttggagggctttgagatctgttttggaggaggagatatcttcatataaaaactacacagaagatttctgaggaacatctttgtgaattgtgcacctaagtcacagtgttgaacctatcttttgattcagcagttttgaatc

3’:atctccctttttgtagaatctgcaagtgaatatttggggccctattttgccctatattggaaaaggaaatatcttcaaatagaaactacacagaagcattctgagaaactactctgtgatgcgtgcattcatctcacagggtacaacctttctttggattgagcagttttgaaacactctttttgtagaatctgcaagtggatatttagagtgatttgaggcctattgtgtaaagggaaatttcttcaactaaaaactacccagaagcattctgagaaacttctttgtgatctgtgcattcatctcacagagttggatgtttctattgatacagcagttttgaaacactctttttttagaatctgtaaatggatatttggagtcttttcaggcctacagtgtagaaggaaatatcttcacataaaaactatgcagaagcattcggcaaaacttgtttgtgatgtgtgcattcatctcacagagttgaatgtctctgttgattgagcagttttgaaacactctttttgtagaatctgcaagtggatatttggagctcattggggcctactgtggaaaaacagataacttctcataaaaactacacagaagcattctgagaaacatctttgtgagttgtgcactgaagtgacagtgttgaacctatcttttgattcagcagttttgagtctctctttttacagaatctgagagtggatatttggagcgctttgaggcgcactgtggaaaatgaaatatcttcacacaaaaactacacagaagcattctgagaaacatctttgtgaggtgtgcactgaagtcacattgttgaacctatgtttt

F: GAACCTATCTTTTGATTCAGCAGT

R: ACAGTAGGCCCCAATGAGCT

LENGTH:409

**Supplementary Table 3. Distribution and amount of unique eccDNAs/ecDNAs on each chromosome**

| **chromosome** | **unique eccDNAs** | **unique ecDNAs** |
| --- | --- | --- |
| 1 | 113 | 3 |
| 2 | 137 | 4 |
| 3 | 81 | 6 |
| 4 | 84 | 7 |
| 5 | 145 | 7 |
| 6 | 83 | 7 |
| 7 | 121 | 17 |
| 8 | 73 | 6 |
| 9 | 64 | 3 |
| 10 | 87 | 4 |
| 11 | 87 | 5 |
| 12 | 93 | 9 |
| 13 | 71 | 0 |
| 14 | 56 | 5 |
| 15 | 47 | 4 |
| 16 | 48 | 6 |
| 17 | 193 | 67 |
| 18 | 48 | 3 |
| 19 | 69 | 6 |
| 20 | 95 | 23 |
| 21 | 25 | 0 |
| 22 | 37 | 2 |
| MT | 1 | 0 |
| X | 85 | 7 |
| Y | 38 | 6 |

**Supplementary Table 4. Distribution of eccDNAs/ecDNAs in the indicated genomic regions.**

| **Type** | **FGR** (eccDNA/ecDNA) | | **Nor**(eccDNA/ecDNA) | |
| --- | --- | --- | --- | --- |
| 5'UTR | 2.7 | 32.37 | 1.53 | 39.17 |
| Coding-Exon | 3.67 | 35.97 | 3.62 | 42.5 |
| ExonPlus | 7.02 | 40.29 | 5.95 | 48.33 |
| Introns | 46.54 | 40.29 | 47.14 | 48.33 |
| 3'UTR | 3.24 | 35.97 | 2.01 | 42.5 |
| Genic.2000bpDown | 4.54 | 40.29 | 3.38 | 48.33 |
| Genic.2000bpUp | 4.54 | 39.57 | 3.86 | 47.5 |
| Genic | 48.38 | 40.29 | 49.16 | 48.33 |
| CpG.2000bpDown | 26.03 | 66.91 | 26.79 | 73.33 |
| CpG.2000bpUp | 25.05 | 66.19 | 26.39 | 71.67 |
| CpG | 15.01 | 66.19 | 18.42 | 72.5 |
| Transposon | 57.24 | 81.29 | 61.38 | 84.17 |
| Alu | 23.54 | 76.98 | 25.66 | 76.67 |

**Supplementary Table 5. The overlapping between circRNAs and ecDNA00000164**

| **ecDNA_id** | **ecDNA**  **pos** | **ecDNA_**  **overlap (%)** | **circRNA_id** | **circRNA_**  **pos** | **circRNA_**  **overlap (%)** |
| --- | --- | --- | --- | --- | --- |
| EcDNA  00000164 | 6:57345464-60256901 | 100 | hsa_circ_  0076892 | 6:57317623-  60430572 | 93.5267 |
| EcDNA  00000164 | 6:57345464-60256901 | 100 | hsa_circ_  0076893 | 6:57317623-  60545629 | 90.193 |
| EcDNA  00000164 | 6:57345464-60256901 | 100 | hsa_circ_  0076895 | 6:57318436-  60430572 | 93.5511 |
| EcDNA  00000164 | 6:57345464-60256901 | 100 | hsa_circ_  0076896 | 6:57318436-  60504694 | 91.3748 |
| EcDNA  00000164 | 6:57345464-60256901 | 100 | hsa_circ_  0076897 | 6:57320456-  60504694 | 91.4328 |
| EcDNA  00000164 | 6:57345464-60256901 | 100 | hsa_circ_  0076899 | 6:57324200-  60425439 | 93.8798 |
| EcDNA  00000164 | 6:57345464-60256901 | 98.8172 | hsa_circ_  0002709 | 6:57379900-  60430572 | 94.3071 |
| EcDNA  00000164 | 6:57345464-60256901 | 98.8172 | hsa_circ_  00076900 | 6:57379900-  60504694 | 92.0701 |
